# Use of Novel Homochiral Thioureas Camphor Derived as Asymmetric Organocatalysts in the Stereoselective Formation of Glycosidic Bonds

**DOI:** 10.3390/molecules29040811

**Published:** 2024-02-09

**Authors:** Mildred López, Gabriela Huelgas, Mario Sánchez, Adalid Armenta, Angel Mendoza, José Daniel Lozada-Ramírez, Cecilia Anaya de Parrodi

**Affiliations:** 1 Departamento de Ciencias Químico-Biológicas, Universidad de las Américas Puebla, Puebla 72810, Mexico; mildred.lopezvz@udlap.mx (M.L.); gabriela.huelgas@udlap.mx (G.H.); jose.lozada@udlap.mx (J.D.L.-R.); 2 Centro de Investigación en Materiales Avanzados S.C., Alianza Norte 202, PIIT, Apodaca 66628, Mexico; mario.sanchez@cimav.edu.mx (M.S.); adalid.torres@cimav.edu.mx (A.A.); 3 Centro de Química, Instituto de Ciencias, Benemérita Universidad Autónoma de Puebla, Puebla 72570, Mexico; angel.mendoza@correo.buap.mx

**Keywords:** camphor, stereoselectivity, glycosylation, thiourea, asymmetric, organocatalyst, solvent free

## Abstract

We synthesized six new camphor-derived homochiral thioureas **1**–**6**, from commercially available (1*R*)-(−)-camphorquinone. These new compounds **1**–**6** were evaluated as asymmetric organocatalysts in the stereoselective formation of glycosidic bonds, with 2,3,4,6-tetra-*O*-benzyl-D-glucopyranosyl and 2,3,4,6-tetra-*O*-benzyl-D-galactopyranosyl trichloroacetimidates as donors, and several alcohols as glycosyl acceptors, such as methanol, ethanol, 1-propanol, 1-butanol, 1-octanol, *iso*-propanol, *tert*-butanol, cyclohexanol, phenol, 1-naphtol, and 2-naphtol. Optimization of the asymmetric glycosylation reaction was achieved by modifying reaction conditions such as solvent, additive, loading of catalyst, temperature, and time of reaction. The best result was obtained with 2,3,4,6-tetra-*O*-benzyl-D-galactopyranosyl trichloroacetimidates, using 15 mol% of organocatalyst **1**, in the presence of 2 equiv of MeOH in solvent-free conditions at room temperature for 1.5 h, affording the glycosidic compound in a 99% yield and 1:73 α:β stereoselectivity; under the same reaction conditions, without using a catalyst, the obtained stereoselectivity was 1:35 α:β. Computational calculations prior to the formation of the products were modeled, using density functional theory, M06-2X/6-31G(d,p) and M06-2X/6-311++G(2d,2p) methods. We observed that the preference for β glycoside formation, through a stereoselective inverted substitution, relies on steric effects and the formation of hydrogen bonds between thiourea **1** and methanol in the complex formed.

## 1. Introduction

**Figure 1:**
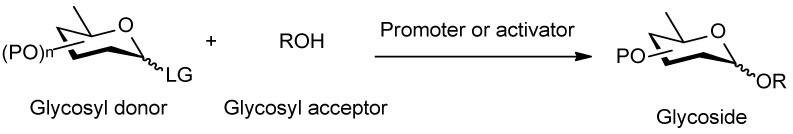
Glycosylation reaction.

Biologically functional carbohydrates have diverse structures, and it is necessary to obtain homogeneous and well-defined materials for their study. The isolation and purification of such natural carbohydrates implies laborious steps, therefore synthetic organic chemistry has been identified as a potential tool to prepare complex oligosaccharides [1]. Glycosylation reactions are fundamental processes for joining monosaccharides, which are essential in the synthesis of oligosaccharides. This process involves the displacement of a leaving group from a glycosyl donor to a glycosyl receptor, generally aimed at a promoter or activator (Figure 1) [2]. These reactions are influenced by various factors affecting its result, including solvent [3], temperature [4], reactant concentration [5], activation method [6], chemical, stereo, and electronic properties of the donor and acceptor [7].

**Scheme 1:**
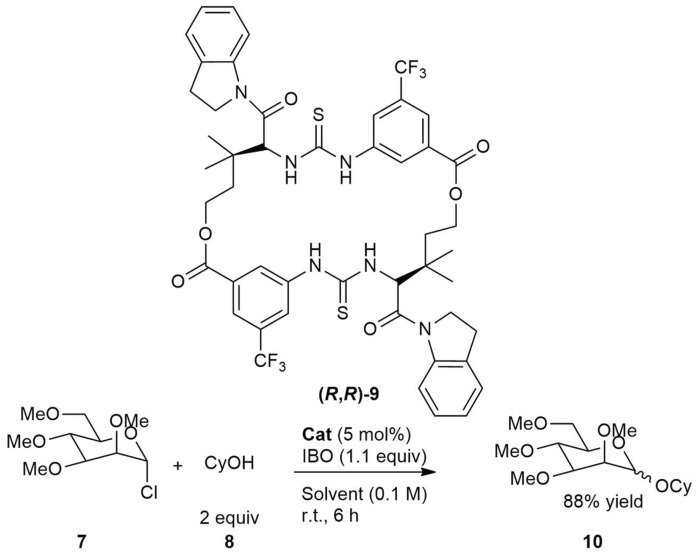
Glycosylation reaction catalyzed by a macrocyclic bis-thiourea **9**.

Among the most commonly used donors are glycosyl iodides [8], thioglycosides [3], glycosyl thioimidates [9], carboxybenzyl glycosides [10,11], and trichloroacetimidates [12]. The trichloroacetimidates have been suitable glycosyl donors due to their easy preparation in basic media and moderate chemical stability at room temperature. In the last two decades, there has been an enormous growth in the development of new glycosylation methodologies, whose efficiency have been generally evaluated by measuring chemical yield, regioselectivity, and stereoselectivity α/β [13,14,15,16,17,18]. Despite numerous advances throughout the last few years, there is not a universal protocol that permits the formation of glycosidic bonds and the main challenge is stereoselectivity [19]. Glycosylation methodologies can be classified depending on the promoter or activator into four different groups as follows [20]: transition metals catalysis [21], photochemical reactions [13], reactions in the presence of designed modulators to control selectivity [22], and reactions with organocatalysts [16,23,24,25,26,27,28,29]. Organocatalysts as activators are environmentally friendly and are ease functionalized. Organocatalytic glycosylation can be classified into three principal groups based on the type of catalyst used as follows: Brønsted acids, organoboron, and thioureas [30]. Thioureas, as organocatalysts, offer important advantages, because they act as donors of hydrogen bonds and weak acids resulting in higher tolerance to several functional groups, chemoselectivity, and stereocontrol. These kinds of catalyst are synthesized through construction blocks that are commonly available and easily modifiable; usually the groups attached to N can be changed to syntonize the organocatalyst. The electronic nature of thiourea is also modified with the insertion of chiral components [30]. Park et al. designed macrocyclic bis-thioureas (*R*,*R*)-**9**, which catalyzed the stereospecific inverted substitution using glucosyl chlorides as the donor and cyclohexanol as the acceptor, affording an 88% yield of the glycosylation product at rt for 6 h, adding IBO and solvent (Scheme 1) [26].

**Scheme 2:**
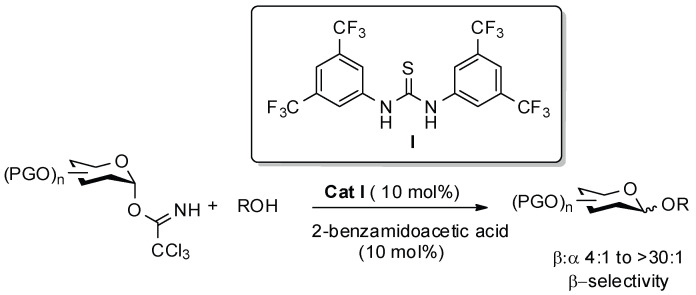
Glycosylation reaction co-catalyzed by achiral thiourea **I**.

In 2013, Geng et al. presented the cooperative catalysis of glycosylation reactions using *O*-trichloroacetimidate donors [25]. Specifically, thiourea as co-catalysts exhibit a cooperative performance with a strong effect on reaction velocity, yield, and selectivity of glycosylation reactions. Dubey et al., (2019) demonstrated a fast glycosylation method, which is highly selective and efficient for preparing β-glycosides using a cooperative promoter system of *N*-benzoylglycine/thiourea (I) [31]. The reaction is highly applicable to a variety of glycosyl donors, and the nucleophile receptors proceed with high selectivity and a high yield, tolerating the most common protective groups. These reaction conditions can be an attractive alternative to existing procedures. Achiral thiourea, as a co-catalyst, exhibits a cooperative performance, having a strong effect on the reaction velocity, yield, and selectivity of glycosylation (Scheme 2).

Solvent-free methodologies have been developed recently in stereoselective glycosylation reactions [32]; however, these procedures are uncommon in carbohydrate chemistry and have only directed very few applications to glycosylation reactions [33]. Solvent-free reactions avoid the use of polluting, high-boiling, toxic, and commonly used organic solvents; such approaches allowed a great simplification and improvement [34,35].

Meanwhile, camphor is a privileged chiral construction block in asymmetric catalysis because of its availability in both enantiomeric forms; additionally, it can suffer an ample variety of chemical transformations [36], allowing the synthesis of compounds with diverse structures and functionalities [37,38].

Herein, we report the synthesis and application of homochiral thioureas **1**–**6**, using camphor as the construction skeleton. We evaluated them in the stereoselective glycosylation reaction, which has been optimized using several reaction conditions. We performed computational calculations to validate the preference of the formation of β-glycosylation product.

## 2. Results and Discussion

### 2.1. Synthesis of New Homochiral Thioureas Derived from Camphor as Potential Organocatalysts

**Figure 2:**
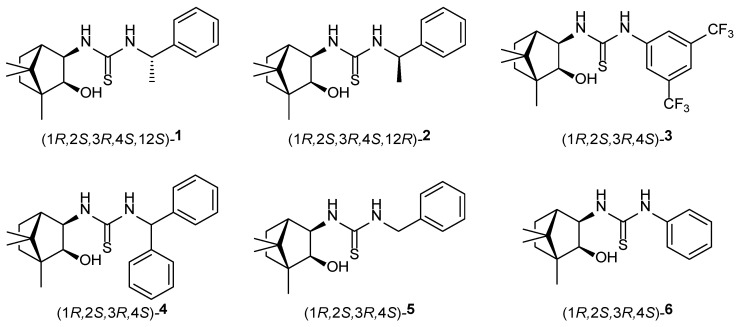
New homochiral thioureas camphor-derived **1**–**6**.

Homochiral thioureas camphor-derived **1**–**6** were prepared following the methodology reported by Santacruz et al. [39]. Diverse structural motives have been introduced at the nitrogen atom of the thiourea. Alongside them, we introduce radicals, which might influence the stereoselectivity of the glycosylation reaction, such as additional chiral centers, (*S*)-1-methylbenzyl- and (*R*)-1-methylbenzyl-,1 and 2, respectively, an electro-attractor group, (3,5-trifluoromethyl) phenyl-3, and bulky groups, benzhydryl-, benzyl-, and phenyl-,4,5 and 6, respectively (Figure 2).

**Scheme 3:**
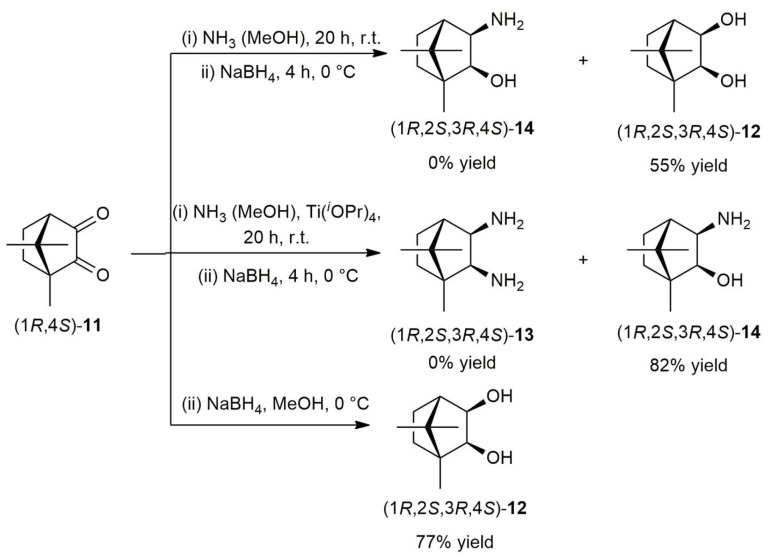
Synthesis of diol **12** and aminoalcohol **14**.

The homochiral thioureas **1**–**6** were synthesized from (1*R*)-(−)-camphorquinone **11** in two steps from aminoalcohol **14**. The synthesis of diamine **13** and aminoalcohol **14** were reported by Periasamy et al. [40]. Following the reaction conditions described in the literature, (1*R*)-(−)-camphorquinone **11** reacted with ammonia (1 M in MeOH), which was followed by a reduction with NaBH_4_, affording diol **12** in a low 55% yield, but aminoalcohol **14** was not obtained (Scheme 3). Based on these results, we prepared diol **12** by direct reduction with NaBH_4_ affording a 77% yield in a moderate yield (Scheme 3). As stated in the literature, we proceeded with the reaction of (1*R*)-(−)-camphorquinone **11** with ammonia (1 M in MeOH) and titanium tetraisopropoxide, followed by a reduction with NaBH_4_, affording only aminoalcohol **14** in an 82% yield, but diamine **13** was not observed (Scheme 3) [40].

Using aminoalcohol **14**, we prepared homochiral thioureas **1**–**6** in CH_2_Cl_2_ as a solvent for 2 h at room temperature in the presence of several isothiocyanates (1.1 equiv) as follows: (*S*)-1-methylbenzyl isothiocyanate, (*R*)-1-methylbenzyl isothiocyanate, (3,5-trifluoromethyl)-phenyl isothiocyanate, benzhydryl isothiocyanate, benzyl isothiocyanate, and phenyl isothiocyanate. The products were purified by flash chromatography with basic silica (triethylamine/SiO_2_ = 2.0% *v*/*w*) and hexane/ethyl acetate (4:1 *v*/*v*) as the eluent. Thioureas **1**–**6** were isolated in moderate to good yields (64–93%, Table 1). The two steps’ synthesis afforded overall yields in the range of 52–76%.

**Figure 3:**
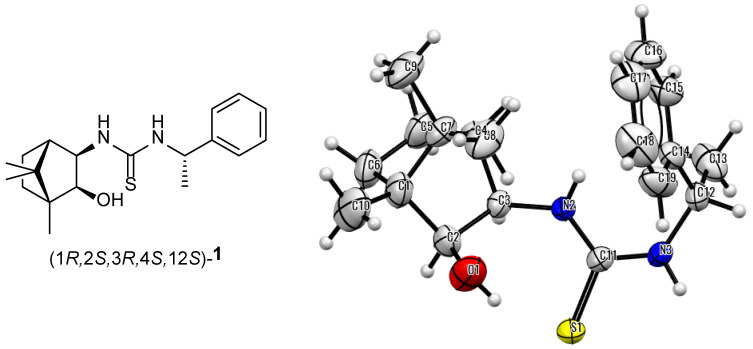
Solid-state structure of 1-((1*R*,2*S*,3*R*,4*S*)-2-Hydroxy-1,7,7-trimethylbicyclo[2.2.1]heptan-3-yl)-3-((*S*)-1-phenylethyl)thiourea.

**Figure 4:**
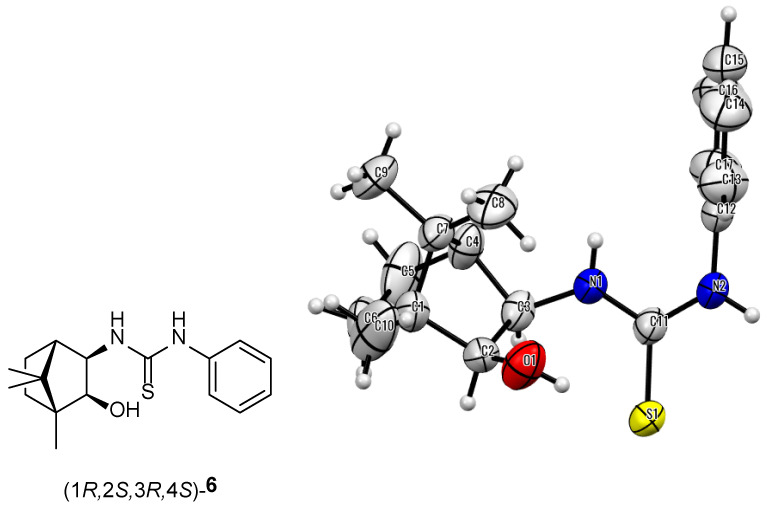
Solid-state structure of 1-((1*R*,2*S*,3*R*,4*S*)-2-Hydroxy-1,7,7-trimethylbicyclo[2.2.1]heptan-3-yl)-3-phenylthiourea.

All camphor-derived thioureas **1**–**6** were fully characterized. Compounds (1*R*,2*S*,3*R*,4*S*,12*S*)-**1** and (1*R*,2*S*,3*R*,4*S*)-**6** gave suitable crystals for X-ray diffraction analysis. Representations of molecular structures are shown in Figure 3 and Figure 4.

### 2.2. Homochiral Thioureas ***1**–**6*** as Organocatalysts in Glycosylation Reaction

Once compounds **1**–**6** were synthesized, we investigated the potential of homochiral thioureas as organocatalysts in the stereoselective glycosylation reaction. For the optimization of the reaction conditions, we performed the glycosylation reaction of 2,3,4,6-tetra-*O*-benzyl-D-galactopyranosyl trichloroacetimidate **15 [28]** (1 equiv) as the glycosyl donor and 2 equiv of methanol as the glycosyl acceptor in the presence of organocatalysts **1** (Table 2), modifying conditions such as the following: solvent, temperature, organocatalyst loading, and additives. 

First, we decided to use several polar aprotic solvents such as CH_3_CN, CH_2_Cl_2_, and THF. In all cases, the glycosylation reaction afforded the product after 120 h, in poor to low yields (15–40%), favoring selective inversion of the configuration of the anomeric center in the range of 1:8 to 1:13 (α/β) (Table 2, entries 1–3). 

Second, the reaction was performed in the presence of non-polar aprotic solvents such as ethyl ether, tert-butyl methyl ether, and toluene. Ethyl ether and tert-butyl methyl ether after 120 h afforded the expected product in poor to low yields (12–47%), favoring selective inversion of the configuration of 1:12 and 1:15 (α/β), respectively (Table 2, entries 4 and 5). The reaction did not proceed to reveal the presence of toluene as solvent (Table 2, entry 6). 

Based on these results, we decided to perform the reaction in solvent-free conditions using no added solvent except for 2 equiv of MeOH, which is polar and protic. The reaction finished after only 1.5 h, affording a 99% yield and 1:73 α/β, i.e., circa 99% stereospecific inverted substitution product (Table 2, entry 7). 

The following experiments were centered on examining the influence of lower temperatures (Table 2, −25 and 0 °C, entries 8 and 9, respectively), affording the product in a lower yield and lower selectivity compared to room temperature. We observed that the reaction time was longer as the temperature diminished (Table 2, 24 and 2 h, entries 8 and 9, respectively). 

Then, we proceeded to determine the influence of catalyst loading on selectivity. We modified the amount of organocatalyst **1** from 15 mol% to 5, 10, and 20 mol%, and achieved the best results with 15 mol% of catalyst loading (Table 1, entries 10–12). 

Finally, potassium carbonate and molecular sieve were tested as additives. The product was only formed in the presence of molecular sieve [26] at room temperature, even though selectivity decreased compared to the reaction without additive (Table 2, entries 13–14).

Once we optimized the reaction conditions, the glycosylation reaction was carried out without the presence of a catalyst using the best reaction conditions (Table 2, entry 7), resulting in a selectivity of 1:35 α:β, which was achieved because of the chirality of galactopyranosyltrichloroacetimidate (Table 3, entry 1). Subsequently, we evaluated the remaining homochiral thioureas. Thioureas **2**–**6** afforded the glycosylation product in 81 to 99% yield, with a higher selectivity of β-glycoside as the major product 1:42 to 1:68 α/β (Table 3, entries 3–7), proving that all thioureas have the capacity to catalyze the stereoselective glycosylation reaction with the inversion of the configuration of the anomeric center. Organocatalyst **2** is the mismatch diastereomer of organocatalyst **1**, i.e., it had the lowest performance with an 81% yield and 1:42 α/β selectivity, and organocatalyst **1** gave the best stereoselectivity of all the thioureas.

We explored the scope of this methodology performing the reaction with 2,3,4,6-tetra*-O-*benzyl-α-D-glucopyranosyl trichloroacetimidate **17** [41] as the glycosyl donor (1 equiv) and with MeOH as the glycosyl acceptor (2 equiv), in solvent-free reaction conditions, catalyzed by 15 mol% of thioureas **1**–**6**. The reaction was followed by TLC until the total consumption of raw material, affording the stereoselective glycosylation product with moderate to high yields (58–92%) and β-selectivity from low to high (1:6 to 1:58). On the one hand, thiourea **1** showed the highest efficiency, with an 81% yield and 1:58 α/β stereoselectivity (Table 4, entry 1). On the other hand, organocatalyst **2** gave the worst result, i.e., the mismatched diastereomer of compound **1**, affording a 58% yield and 1:6 β-selectivity (Table 4, entry 2). Thioureas **3**–**6** afforded the glycosylation product in good yields (83–92%) and low to high β-selectivity 1:12–1:57 α/β (Table 4, entries 3–6).

To explore the scope of this methodology, we also performed the reaction in the optimized conditions using 2,3,4,6-tetra-*O*-benzyl-D-galactopyranosyl trichloroacetimidate **15** [28] (1 equiv) as the glycosyl donor with 2 equiv of several acceptors such as ethanol, 1-propanol, 1-butanol, 1-octanol, *iso*-propanol, *tert*-butanol, cyclohexanol, phenol, 1-napthol, and 2-naphtol. We observed the formation of the glycosylation products in all cases. Methanol showed the highest β-selectivity due to the lowest steric hindrance (Table 5, entry 1). It is important to note that β-selectivity decreases as the carbon chain is larger (Table 5, entries 2–5) or the bulkiness of the carbon chain is bigger (Table 5, entries 6–7). We observed that in the cases of 1-propanol and 1-butanol, the reaction occurred almost to completion in 4 h (Table 5, 95 and 96%, entries 3 and 4, respectively), and in the case of 1-octanol, the reaction was carried out in 3 h with a yield of 76% (Table 5, entry 5). When cyclohexanol was employed, a significant decrease in yield was observed, resulting in an α:β ratio of 1:3 in 4 h (Table 5, 25%, entry 8). When solid alcohols such as phenol, 1-naphthol, and 2-naphthol were utilized, the reactions were solubilized in TBME, a solvent that yielded the best result in previous studies (Table 2, entry 5). With phenol, a 1:1.3 α:β ratio was achieved at a 39% yield (Table 5, entry 9). Finally, with 1-naphthol and 2-naphthol, an increase in reaction time was observed (6 h), leading to the formation of the anomeric mixture in low yields (Table 5, entries 10 and 11).

**Figure 5:**
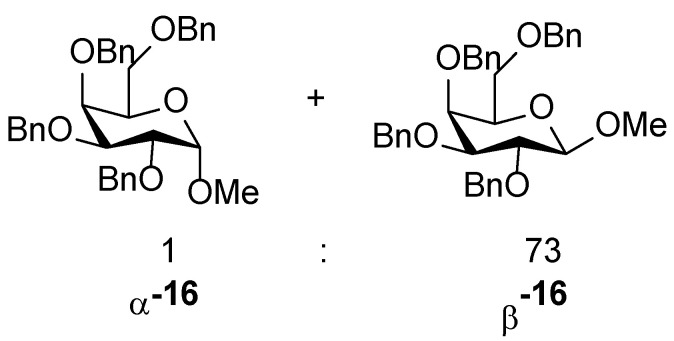
Glycoside structures in α and β ratios.

To explain the formation of a 1:73 ratio (α:β) that favors product β-**16**, related to α-**16** (Figure 5), we modeled the possible interaction between the three structures involved in the reaction, i.e., the donor, acceptor, and organocatalyst, before the formation of β-**16**.

**Figure 6:**
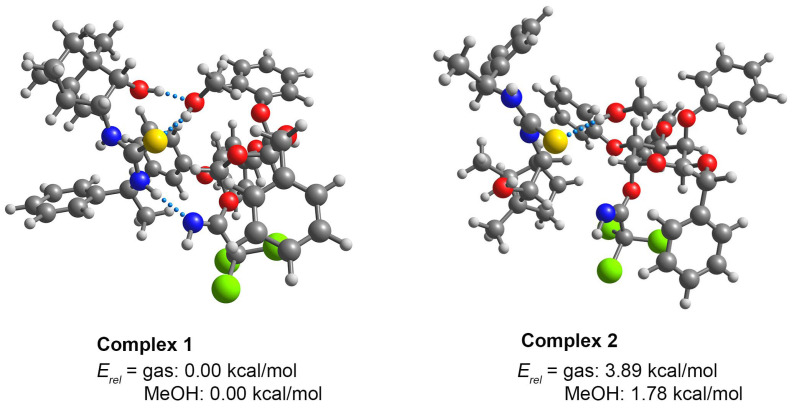
Relative energies of complexes **1** and **2** in the gas phase and methanol as solvent. Color codes were used to identify different atom types as follows: dark gray for carbon (C), red for oxygen (O), light gray for hydrogen (H), yellow for sulfur (S), green for chlorine (Cl), and blue for nitrogen (N). The blue dots represent weak interactions known as hydrogen bonds.

First, we look for the lowest energy local minimum for the complex involving the three species 2,3,4,6-tetra-*O*-benzyl-D-galactopyranosyl trichloroacetimidate **15**, MeOH, and organocatalyst **1**. Computational calculations have suggested two structures with the lowest energy, both of which are stabilized by various hydrogen bonds (Figure 6). The orientation of the organocatalyst **1** is the difference in both complexes. Optimizations were performed using the M06-2X/6-31G(d,p) method and all atoms were considered without any restriction of motion or geometry. Energies were computed using the M06-2X/6-311++G(2d,2p) method [42,43]. All calculations were performed using Gaussian 16 [44]. The IEFPCM solvation model [45] was used and methanol was the solvent.

**Figure 7:**
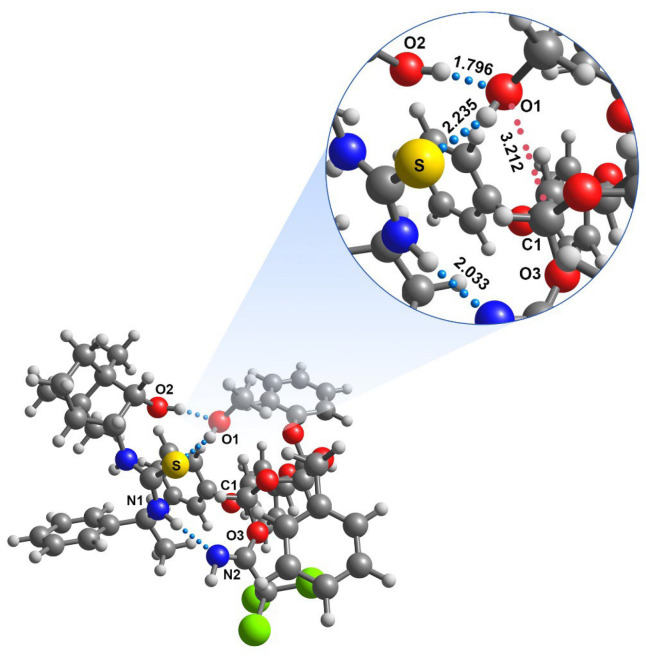
Hydrogen bonds in complex **1** and their distances (Å). The orientation of methanol toward C1 is also represented. Each type of atom is indicated by specific colors, namely dark gray (C), red (O), light gray (H), yellow (S), green (Cl), and blue (N). The blue dots represent weak interactions known as hydrogen bonds.

Complex **1** is stabilized by three hydrogen bonds, two of the type O--H--O and one O--H--S, while complex **2** only shows a hydrogen bond, O--H--S. This explains why complex **1** has the lowest energy. Also, these structures were modeled in the presence of a solvent in order to know the effect of methanol on the energies; the result was a decrease in the difference between the values of the energies, but it was confirmed that complex **1** is more stable. Figure 7 shows the internal interactions of the hydrogen bonds and O1--C1 (orange dotted line) distances. Methanol O1 has a good orientation to attack the C1 of the six-membered heterocycle because of less steric agglomeration observed in that position (Figure 7). This O1--C1 distance has a value of 3.212 Å. In addition, different internal hydrogen bonds that stabilize the geometry can be observed in more detail.

**Figure 8:**
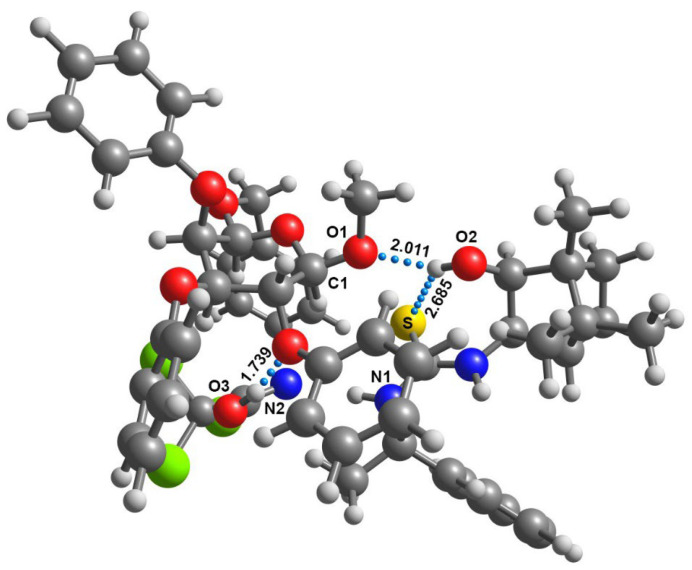
Complex **3** structure, which is stabilized by three main hydrogen bonds. Color code for each atom is as follows: dark gray (C), red (O), light gray (H), yellow (S), green (Cl), and blue (N). The blue dots represent weak interactions known as hydrogen bonds.

The preference for β-glycoside formation relies on steric effects and the formation of hydrogen bonds (complex **1**), which help in stabilization. The final product was modeled as complex **3** (Figure 8). This molecular complex is stabilized by three main hydrogen bonds, two in which hydrogen participates in O2 with methanol, oxygen, and sulfur, and the third is formed by the exiting fragment of C1 with the oxygen of the -OPh group. With the prepared models, it is evident that the formation of complexes **3** and **2** is favored by steric effects because the attack is performed opposite to O3, which is less sterically hindered.

**Figure 9:**
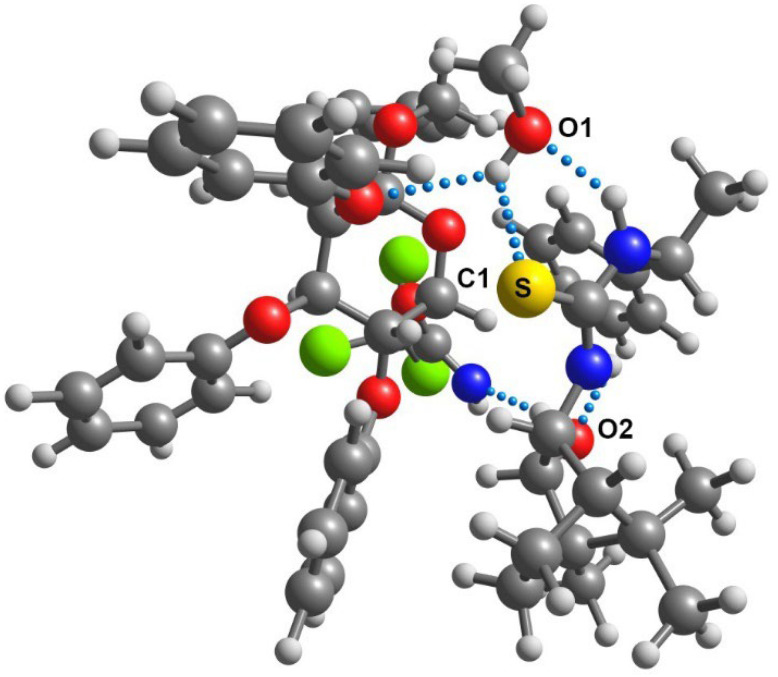
Electronic structure for complex **4**, with organocatalyst **2**. Color code for each atom is as follows: dark gray (C), red (O), light gray (H), yellow (S), green (Cl), and blue (N). The blue dots represent weak interactions known as hydrogen bonds.

Finally, to understand the poor effectiveness of organocatalyst **2** (mismatch diastereomer of thiourea **1**), complex **4** has been modeled, in which it can be observed that the entry of methanol is stereoelectronically impeded by the thiourea group; moreover, this complex is stabilized, similar to complex **1**, by multiple hydrogen bonds (Figure 9). It is important to note that in this complex, the distance O1-C1 is much longer with a value of 4.026 Å.

## 3. Materials and Methods

All chemicals were obtained from commercial suppliers and used without purification. Liquid chemicals, solutions, or solvents were added using a syringe (mL) or a micropipette (µL). Reactions were followed by thin-layer chromatography using silica gel 60 F_254_ plates supported in aluminum (Merck, Rahway, NJ, USA, Telos) and were revealed by UV light (254 nm) using phosphomolybdic acid in ethanol (0.25 g/mL), or with iodide vapors. The products obtained were concentrated using Rotavapor Büchi R-300 (bath temperatures above 40 °C) and reduced pressure depending on the solvent used.

^1^H and ^13^C NMR spectra were obtained in CDCl_3_ using a Bruker (Billerica, MA, USA) AscendTM (400 MHz) spectrometer with tetramethylsilane (TMS) as a reference at 0 ppm. Optical rotation recordings were measured using an Autopol III (Rudolph Research Analytical, Hackettstown, NJ, USA) polarimeter at 20 °C and 589 nm, using CHCl_3_ as the blank and solvent, and concentrations were calculated in g/mL. Infrared (IR) spectra were obtained using an FTIR Cary 630 spectrophotometer (Agilent Technologies, Santa Clara, CA, USA).

Single-crystal XRD analysis was performed as a single crystal of each compound was mounted on a loop plastic fiber. Diffraction analyses were carried out on an Oxford Diffraction Gemini “Atlas” diffractometer, Agilent Technologies (Oxford, UK), equipped with a charge-coupled device area detector, sealed X-ray tube (λMoKα = 0.71073 Å), and a graphite monochromator. The CrysAlis PRO (v38.46) and CrysAlis RED (Version 1.171.35.11) software packages were used for data collection and integration. The collected data were corrected for absorbance using an analytical numerical correction [46] with a multifaceted crystal model. Structure solution and refinement were carried out using Olex2 software [47]. To prepare the material for publication, Mercury 4.0 and Olex2 software were used [48]. 

### 3.1. General Synthesis of Homochiral Thioureas

To a solution of the chiral aminoalcohol [40] (1*R*,2*S*,3*R*,4*S*)-**14** (1 equiv) in CH_2_Cl_2_ (10 mL) were added (*S*)-1-methylbenzyl isothiocyanate, (*R*)-1-methylbenzyl isothiocyanate, (3,5-trifluoromethyl) phenyl isothiocyanate, benzhydryl isothiocyanate, benzyl isothiocyanate, and phenyl isothiocyanate (1.1 equiv) [39] under a nitrogen atmosphere. The mixture was stirred at room temperature for 2 h, then the solvent was eliminated by reduced pressure and the products obtained were purified using a chromatographic column with basic silica (triethylamine/SiO_2_ = 2.0% *v*/*w*, hexane/ethyl acetate 4:1 *v*/*v*) to obtain thioureas **1**–**6**.

#### 3.1.1. Synthesis of 1-((1*R*,2*S*,3*R*,4*S*)-2-Hydroxy-1,7,7-Trimethylbicyclo[2.2.1]Heptan-3-yl)-3-((*S*)-1-Phenylethyl)Thiourea-**1**

Compound **1** was synthesized from aminoalcohol (1*R*,2*S*,3*R*,4*S*)-**14** in the presence of (*S*)-1-methylbenzyl isothiocyanate following the general method. Crystals (79% yield), m.p. 111–113 °C. [α]_D_ = +90.08 (c = 0.086, CHCl_3_). ^1^H NMR (400 MHz, CDCl_3_, δ): 0.63 (s, 3H), 0.69 (s, 3H), 0.87 (s, 3H), 1.01–1.15 (m, 2H), 1.44 (td, *J* = 2.8, 12.0 Hz, 1H), 1.5 (d, *J* = 8 Hz, 3H), 1.60–1.69 (m, 2H), 2.55 (b, 1H), 3.80–3.82 (m, 1H), 3.95 (t, *J* = 8.0, 4.0 Hz, 1H), 4.64 (b, 1H), 6.04 (b, 1H), 6.39 (b, 1H), 7.26–7.38 (m, 5H). ^13^C NMR (100 MHz, CDCl_3_, δ): 11.2, 20.5, 21.3, 24.2, 25.9, 33.2, 46.8, 49.1, 50.4, 54.1, 63.0, 80.1, 125.7, 128.2, 129.3, 142.0, 180.0 IR-FT: 3334, 3256, 2954, 2873, 2290, 2169, 1700, 1522, 1449, 1407, 1378, 1347, 1318, 1242, 1215, 1123, 1147, 1089, 1054, 963, 814, 748, 696, 664, 607 cm^−1^. HRMS (FAB^+^) *m*/*z* calcd. for [C_19_H_29_ON_2_S]: 333.1917, found 333.1972. Recrystallized from hexanes/CH_2_Cl_2_ (5:1), 0.526 × 0.246 × 0.195 mm^3^, C_19_H_28_N_2_OS (*M* = 332.49 g/mol): monoclinic, P2_1_ (no.4) *a* = 7.6886(3) Å, *b* = 27.9174(10) Å, *c* = 8.9652(4) Å, *β* = 101.610(4)°, *V* = 1884.97(13) Å^3^, *Z* = 4, *T* = 293(2) K, μ = 0.178 mm^−1^, ρ_calcd_ = 1.172 g/cm^3^, 25,311 reflections measured (5.838° ≤ 2Θ ≤ 61.012), [Rint = 0.0581, R_sigma_ = 0.0727]. F(000) = 720.0. Final *R*_1_ was 0.0821 (I > 2σ(I)) and *wR*_2_ was 0.1392 for all data. CCDC deposition number: 2,302,046.

#### 3.1.2. Synthesis of 1-((1*R*,2*S*,3*R*,4*S*)-2-Hydroxy-1,7,7-Trimethylbicyclo[2.2.1]Heptan-3-yl)-3-((*R*)-1-Phenylethyl)Thiourea-**2**

Compound **2** was synthesized from aminoalcohol (1*R*,2*S*,3*R*,4*S*)-**14** in the presence of (*R*)-1-methylbenzyl isothiocyanate following the general method. White solid (90% yield), m.p. 65–67 °C. [α]_D_ = +106.37 (c = 0.014, CHCl_3_). ^1^H NMR (400 MHz, CDCl_3_, δ): 0.60 (s, 3H), 0.71 (s, 3H), 0.83 (s, 3H), 0.99–1.06 (m, 1H), 1.10–1.17 (m, 1H), 1.44 (td, *J* = 4.0, 12.0 Hz, 1H), 1.49 (d, *J* = 4 Hz, 3H), 1.64–1.72 (m, 1H), 1.77 (d, *J* = 4 Hz, 1H), 2.31 (b, 1H), 3.73 (d, *J* = 8.0 Hz, 1H), 3.90 (m, 1H), 4.65 (b, 1H), 6.05 (b, 1H), 6.46 (b, 1H), 7.25–7.38 (m, 5H). ^13^C NMR (100 MHz, CDCl_3_, δ): 11.2, 20.1, 21.3, 24.5, 26.1, 33.2, 47.1, 49.0, 50.4, 54.2, 63.5, 80.4, 125.7, 128.2, 129.3, 141.8, 180.5. IR-FT: 3275, 3048, 2951, 2935, 2100, 1890, 1704, 1520, 1341, 1239, 1052, 924, 758. HRMS (FAB^+^) *m*/*z* calcd. for [C_19_H_29_ON_2_S]: 333.1922 found 333.1978.

#### 3.1.3. Synthesis of 3-[3,5-Bis(Trifluoromethyl)phenyl]-1-[(1*R*,2*S*,3*R*,4*S*)-2-Hydroxy-1,7,7-Trimethylbicyclo[2.2.1]Heptan-3-yl]Thiourea-**3**

Compound **3** was synthesized from aminoalcohol (1*R*,2*S*,3*R*,4*S*)-**14** in the presence of (3,5-trifluoromethyl) phenyl isothiocyanate following the general method. White crystals (64% yield), m.p. 184–186 °C. [α]_D_ = +77.12 (c = 0.024, CHCl_3_), ^1^H NMR (400 MHz, CDCl_3_, δ): 0.73 (s, 3H), 0.84 (s, 3H), 0.88 (s, 3H), 0.99–1.03 (m, 1H), 1.13–1.18 (m, 1H), 1.46 (td, *J* = 4.0, 12.0 Hz, 1H), 1.64–1.69 (m, 1H), 1.94 (d, *J* = 4.0 Hz, 1H), 2.46 (b, 1H), 3.80 (dd, *J* = 4.0, 8.0 Hz, 1H), 4.11 (b, 1H), 7.00 (b, 1H), 7.61 (s, 1H), 7.69 (s, 2H), 8.84 (b, 1H). ^13^C NMR (100 MHz, CDCl_3_, δ): 11.1, 20.9, 21.3, 25.9, 33.0, 46.7, 49.3, 50.2, 62.6, 79.8, 119.3, 122.7 (q, ^1^J_CF_ = 271 Hz, CF_3_), 123.7, 133.2, 179.2. IR-FT: 3155, 3031, 2960, 1622, 1552, 1505, 1467, 1375, 1274, 1170, 1122, 1053, 963, 887, 846, 792, 707, 680 cm^−1^. HRMS (FAB^+^) *m*/*z* calcd. for [C_19_H_23_ON_2_F_6_S]: 441.1357, found 441.1339.

#### 3.1.4. Synthesis of 1-Benzhydryl-3-((1*R*,2*S*,3*R*,4*S*)-2-Hydroxy-1,7,7-Trimethylbicyclo[2.2.1]Heptan-3-yl)Thiourea-**4**

Compound **4** was synthesized from aminoalcohol (1*R*,2*S*,3*R*,4*S*)-**14** in the presence of benzhydryl isothicyanate following the general method. White crystals (79% yield), m.p. 152–154 °C. [α]_D_ = +70.27 (c = 0.023, CHCl_3_). ^1^H NMR (400 MHz, CDCl_3_, δ): 0.50 (s, 3H), 0.63 (s, 3H), 0.78 (s, 3H), 0.89–1.10 (m, 2H), 1.19 (s, 1H), 1.34–1.41 (m, 1H), 1.56–1.63 (m, 1H), 2.38 (b, 1H), 3.72 (d, *J* = 7.2, 1H), 3.90 (b, 1H), 5.66 (b, 1H), 6.09 (b, 1H), 6.44 (b, 1H), 7.19–7.29 (m, 10H). ^13^C NMR (100 MHz, CDCl_3_, δ): 11.4, 20.3, 21.4, 26.0, 33.2, 46.9, 49.1, 50.3, 62.5, 63.1, 79.9, 127.3, 127.4, 128.2, 129.1, 140.0, 180.0 IR-FT: 3675, 3263, 2952, 2928, 2079, 1873, 1737, 1523, 1503, 1305, 1217, 1055, 960, 803, 758, 740, 697 cm^−1^. HRMS (FAB^+^) *m*/*z* calcd. for [C_24_H_31_ON_2_S]: 395.2079 found 395.2220.

#### 3.1.5. Synthesis of 1-Benzyl-3-((1*R*,2*S*,3*R*,4*S*)-2-Hydroxy-1,7,7-Trimethylbicyclo[2.2.1]Heptan-3-yl)Thiourea-**5**

Compound **5** was synthesized from aminoalcohol (1*R*,2*S*,3*R*,4*S*)-**14** in the presence of benzyl isothiocyanate following the general method. White solid (74% yield), m.p. 138–139 °C. [α]_D_ = +104.28 (c = 0.007, CHCl_3_). ^1^H NMR (400 MHz, CDCl_3_, δ): 0.68 (s, 3H), 0.80 (s, 3H), 0.82 (s, 3H), 0.94–1.09 (m, 2H), 1.40 (td, *J* = 4.0, 12.0 Hz, 1H), 1.57–1.66 (m, 1H), 1.75 (d, *J* = 4.0 Hz, 1H), 2.96 (b, 1H), 3.74 (d, *J* = 8.0 Hz, 1H), 3.81 (b, 1H), 4.47 (b, 2H), 6.42 (b, 2H), 7.19–7.27 (m, 5H). ^13^C NMR (100 MHz, CDCl_3_, δ): 11.3, 20.8, 21.4, 26.0, 33.2, 47.0, 48.1, 49.0, 50.2, 62.4, 79.9, 127.4, 128.0, 129.0, 136.7, 180.9. IR-FT: 3256, 2949, 2872, 2730, 1528, 1455, 1343, 1307, 1269, 1238, 1123, 1192, 1092, 1052, 961, 856, 797, 731, 694, 645 cm^−1^. HRMS (FAB+) *m*/*z* calcd. for [C_18_H_27_ON_2_S]: 319.1766, found 319.1836.

#### 3.1.6. Synthesis of 1-((1*R*,2*S*,3*R*,4*S*)-2-Hydroxy-1,7,7-Trimethylbicyclo[2.2.1]Heptan-3-yl)-3-Phenylthiourea **6**

Compound **6** was synthesized from aminoalcohol (1*R*,2*S*,3*R*,4*S*)-**14** in the presence of phenyl isothiocyanate following the general method. White solid (93% yield), m.p. 171–173 °C. [α]_D_ = +106.04 (c = 0.008, CHCl_3_), ^1^H NMR (400 MHz, CDCl_3_, δ): 0.77 (s, 3H), 0.90 (s, 6H), 1.06–1.13 (m, 1H), 1.18–1.26 (m, 1H), 1.50 (td, *J* = 4.0, 12.0 Hz, 1H), 1.70–1.74 (m, 1H), 1.86 (d, *J* = 8.0 Hz, 1H), 2.62 (b, 1H), 3.90 (d, *J* = 8.0 Hz, 1H), 4.14 (t, *J* = 4.0, 8.0 Hz, 1H), 6.64 (d, *J* = 8.0 Hz, 1H), 7.20–7.22 (m, 2H), 7.27–7.31 (m, 1H), 7.39–7.43 (m, 2H), 7.98 (b, 1H) ^13^C NMR (100 MHz, CDCl_3_, δ): 11.3, 20.9, 21.3, 26.1, 33.2, 46.9, 49.3, 50.3, 63.0, 80.3, 125.2, 127.3, 130.0, 136.1, 179.6. IR-FT: 3431, 3392, 3378, 3218, 3105, 2921, 2338, 2099, 1670, 1614, 1535, 1505, 1391, 1244,1053, 923, 839, 788, 693 cm^−1^. HRMS (FAB^+^) *m*/*z* calcd. for [C_17_H_25_ON_2_S]: 305.1609, found 305.1726. Recrystallized from hexanes/CH_2_Cl_2_ (5:1), 0.52 × 0.33 × 0.21 mm^3^, C_17_H_24_N_2_OS (*M* = 304.44 g/mol): monoclinic, P2_1_ (no. 4), *a* = 7.6667(2) Å, *b* = 11.9881(2) Å, *c* = 18.6749(4) Å, *β* = 100.548(2)°, *V* = 1687.39(6) Å^3^, *Z* = 4, T = 293(2) K, μ = 0.193 mm^−1^, *ρcalcd* = 1.198 g/cm^3^, 54,301 reflections measured (6.208° ≤ 2Θ ≤ 61.012°), 10,257 unique [R_int_ = 0.0384, R_sigma_ = 0.0291]. F(000) = 656.0. Final *R*_1_ was 0.0436 ((I > 2σ(I)), *wR*_2_ was 0.1248 for all data. CCDC deposition number was 2,302,045.

### 3.2. General Methodology for Glycosylation Reaction

In a 25 mL flask with magnetic stirring, 1 equiv of trichloroacetimidate and the corresponding organocatalysts (15 mol%) were weighed. Both were dissolved in solvent (2 mL), and the MeOH was added (2 equiv). The reaction mixture was maintained at room temperature. The reaction was completed by adding triethylamine to the flask (0.5 mL) and washing with a saturated solution of NaCl, followed by ulterior extractions with CH_2_Cl_2_ (3 × 20 mL). The organic phase was dried over Na_2_SO_4_, and the solvent was eliminated using reduced pressure. The crude reaction was analyzed using ^1^H NMR to measure the α:β ratio.

### 3.3. General Methodology for Solvent-Free Glycosylation Reaction [32]

The trichloroacetimidate donor and the corresponding organocatalyst were added as a solution in the smallest possible amount of technical grade CH_2_Cl_2_; the flask was heated at the required temperature and the solvent was distilled off. Then, the acceptor (2 equiv) was added and the reaction was followed by TLC until the disappearance of the raw material. The reaction was completed by adding triethylamine to the flask (0.5 mL) and washing with a saturated solution of NaCl, followed by ulterior extractions with CH_2_Cl_2_ (3 × 20 mL). The organic phase was dried over Na_2_SO_4_, and the solvent was eliminated using reduced pressure. The crude reaction was analyzed using ^1^H NMR to measure the α:β ratio.

## 4. Conclusions

Our research group has developed new camphor-derived organocatalysts **1**–**6**, which proved to be effective in the stereoselective glycosylation reaction. We observed the best results with thiourea **1** and worst with thiourea **2**, which is the mismatch diastereomer of thiourea **1** and is due to the chiral center present in the methylbenzyl group. Thioureas **3**–**6** have a similar performance compared to **1**, even though these organocatalysts have structures with important stereo and electronic differences. Glycosylation reaction conditions were optimized, finding that the best yield and selectivity were afforded at room temperature and in solvent-free conditions. We extended the scope of the methodology for the stereoselective glycosylation reaction using methanol as the acceptor with two glycosyl donors 2,3,4,6-tetra-*O*-benzyl-D-galactopyranosyl trichloroacetimidate **15** and 2,3,4,6-tetra-*O*-benzyl-D-glucopyranosyl trichloroacetimidate **17**, observing that **15** gave a higher yield and selectivity than **17** due to less steric hindrance. Our group extended the scope of the methodology using donor **15** in the presence of several alkyl alcohols as acceptors besides methanol, such as the following: 1-propanol, 1-butanol, 1-octanol, *iso*-propanol, *tert*-butanol, cyclohexanol, phenol, 1-naphtol, and 2-naphtol, and observed that methanol was the best acceptor due to less steric hindrance and had the best hydrogen bond formation ability. In the glycosylation reaction described here, we observe that α-imidates provide β-selectivity; therefore, we consider that the organocatalyzed reaction by chiral thioureas **1**–**6** might proceed through a S_N_2 mechanism, where, initially, the acceptor forms an adduct with the catalyst, followed by the activation of the imidate in a stereoselective manner to provide β-glycosides.

Theoretical calculations were performed to explain the formation of β anomer in the glycosylation reaction product with organocatalysts **1** and **2**. Our findings indicate that β anomer is favored with organocatalyst **1** for steric effects and the formation of hydrogen bonds that helped to the stabilization of the glycosylation product, meanwhile thiourea **2** presents the worst performance as an organocatalyst because a stereoelectronic hindrance occurs in the activation complex produced by the chiral center (*R*) of the thiourea. The reaction conditions used are mild and widely applicable to diverse glycosyl donors and acceptors. Nowadays, our group is focused on the improvement of organocatalytic systems that might be applicable to a larger scope. 

## Figures and Tables

**Table 1:** Synthesis of camphor-derived homochiral thioureas from aminoalcohol **14**.

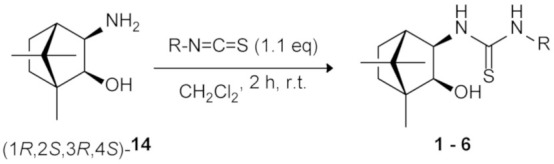			
Entry	R-N=C=S	Product	Yield ^a^ (%)
1	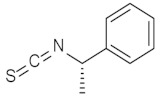	(1*R*,2*S*,3*R*,4*S*,12*S*)-**1**	79
2	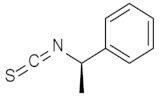	(1*R*,2*S*,3*R*,4*S*,12*R*)-**2**	90
3	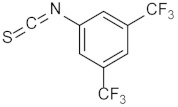	(1*R*,2*S*,3*R*,4*S*)-**3**	64
4	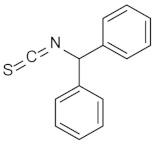	(1*R*,2*S*,3*R*,4*S*)-**4**	79
5	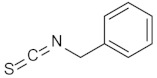	(1*R*,2*S*,3*R*,4*S*)-**5**	74
6	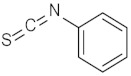	(1*R*,2*S*,3*R*,4*S*)-**6**	93

^a^ Yield determined after purification by flash silica gel chromatography.

**Table 2:** Optimization of selective glycosylation reaction of 2,3,4,6-tetra-*O*-benzyl-D-galactopyranosyl trichloroacetimidate **15** and MeOH using organocatalyst **1** ^a^.

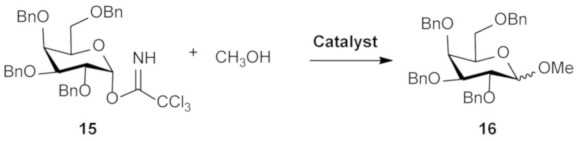							
Entry	Catalyst (mol %)	Additive (2 Equiv)	Solvent (2 mL)	Time (h)	T (°C)	Yield ^b^ (%)	α:β ^c^
1	**1** (15)	None	CH_3_CN	120	r.t.	15	1:8
2	**1** (15)	None	CH_2_Cl_2_	120	r.t.	40	1:13
3	**1** (15)	None	THF	120	r.t.	25	1:8
4	**1** (15)	None	Et_2_O	120	r.t.	12	1:12
5	**1** (15)	None	TBME ^d^	120	r.t.	47	1:15
6	**1** (15)	None	Toluene	120	r.t.	n.r.	-
7	**1** (15)	None	solvent free	1.5	r.t.	99	1:73
8	**1** (15)	None	solvent free	2	0	90	1:38
9	**1** (15)	None	solvent free	24	−25	70	1:41
10	**1** (5)	None	solvent free	1.5	r.t.	90	1:40
11	**1** (10)	None	solvent free	1.5	r.t.	86	1:45
12	**1** (20)	None	solvent free	1.5	r.t.	80	1:40
13	**1** (15)	K_2_CO_3_	solvent free	1.5	r.t.	n.r.	-
14	**1** (15)	Molecular sieve	solvent free	1.5	r.t.	92	1:40

^a^ Reaction conditions: Glycosyl donor: 2,3,4,6-tetra-*O*-benzyl-D-galactopyranosyl trichloroacetimidate **15**, (1 equiv), glycosyl acceptor: MeOH (2 equiv). ^b^ Yield determined after purification by flash silica gel chromatography. ^c^ Anomeric ratio was determined by ^1^H NMR spectroscopy. ^d^ TBME (methyl tertbutyl ether). r.t. indicates room temperature and n.t. indicates not reported.

**Table 3:** Stereoselective glycosylation reaction of 2,3,4,6-tetra-*O*-benzyl-d-galactopyranosyl trichloroacetimidate **15** and MeOH using organocatalysts **1**–**6** ^a^.

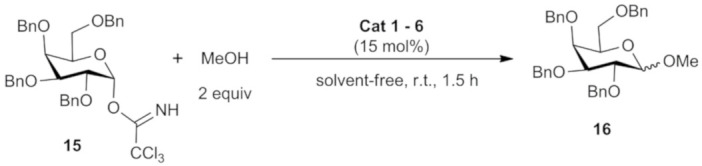			
Entry	Organocatalyst (mol %)	Yield (%) ^b^	α:β ^c^
1	None	82	1:35
2	**1**	99	1:73
3	**2**	81	1:42
4	**3**	99	1:55
5	**4**	95	1:68
6	**5**	99	1:56
7	**6**	93	1:52

^a^ Reaction conditions: Glycosyl donor: 2,3,4,6-tetra-*O*-benzyl-D-galactopyranosyl trichloroacetimidate 15, (1 equiv), glycosyl acceptor: MeOH (2 equiv). ^b^ Yield determined after purification by flash silica gel chromatography. ^c^ Anomeric ratio was determined by ^1^H NMR spectroscopy.

**Table 4:** Stereoselective glycosylation reaction between 2,3,4,6-tetra-*O*-benzyl-α-D-glucopyranosyl trichloroacetimidate **17** and MeOH using organocatalysts **1**–**6** ^a^.

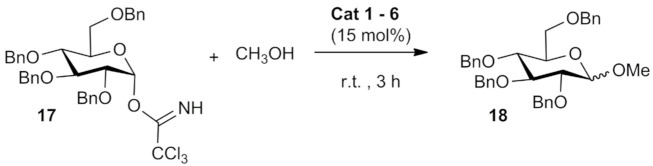			
Entry	Organocatalyst	Yield (%) ^b^	(α:β ratio) ^c^
1	**1**	81	1:58
2	**2**	58	1:6
3	**3**	83	1:14
4	**4**	92	1:53
5	**5**	87	1:12
6	**6**	86	1:57

^a^ Reaction conditions: Glycosyl donor: 2,3,4,6-tetra-*O*-benzyl-α-D-glucopyranosyl trichloroacetimidate **17**, (1 equiv), glycosyl acceptor: MeOH (2 equiv). ^b^ Yield determined after purification by flash silica gel chromatography. ^c^ Anomeric ratio was determined by ^1^H NMR spectroscopy.

**Table 5:** Stereoselective glycosylation reaction between 2,3,4,6-tetra-*O*-benzyl-D-galactopyranosyl trichloroacetimidate **15** and several alcohols using organocatalysts **1** ^a^.

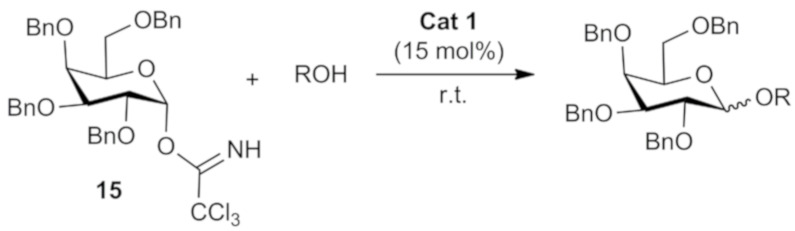				
Entry	ROH	Reaction Time (h)	Yield (%) ^b^	(α:β ratio) ^c^
1	Methanol	1.5	99	1:73
2	Ethanol	1.5	88	1:13
3	1-propanol	4	95	1:10
4	1-butanol	4	96	1:6.5
5	1-octanol	3	76	1:8
6	*iso-*propanol	1.5	52	1:7
7	*tert*-butanol	1.5	66	1:1.1
8	Cyclohexanol	4	25	1:3
9	Phenol ^d^	4	39	1:1.3
10	1-naphtol ^d^	6	26	1:3
11	2-naphtol ^d^	6	13	1:6

^a^ Reaction conditions: Glycosyl donor: 2,3,4,6-tetra-*O*-benzyl-D-galactopyranosyl trichloroacetimidate **15**, (1 equiv), glycosyl acceptor: ROH (2 equiv). ^b^ Anomeric ratio was determined by NMR spectroscopy. ^c^ Yield determined after purification by flash silica gel chromatography. ^d^ Dissolved in 2 mL of TBME (methyl tertbutyl ether).

## Data Availability

Data are contained within the article and Supplementary Materials.

## Supplementary Materials

The following supporting information can be downloaded at https://www.mdpi.com/article/10.3390/molecules29040811/s1, Synthesis and characterization data and NMR for compounds **1**–**6**. X-ray Diffraction data of compounds **1** and **6**. Figure S1. ^1^H NMR of **1**. Figure S2. ^13^C NMR of **1**. Figure S3. HRMS of **1**. Figure S4. ^1^H NMR of **2**. Figure S5. ^13^C NMR **2**. Figure S6. HRMS of **2**. Figure S7. ^1^H NMR of **3**. Figure S8. ^13^C NMR of **3**. Figure S9. HRMS of **3**. Figure S10. ^1^H NMR of **4**. Figure S11. ^13^C NMR of **4**. Figure S12. HRMS of **4**. Figure S13. ^1^H NMR of **5**. Figure S14. ^13^C NMR of **5**. Figure S15. HRMS of **5**. Figure S16. ^1^H NMR of **6**. Figure S17. ^13^C NMR of **6**. Figure S18. HRMS of **6**. Table S1. Crystal data and structure refinement for **1**. Table S2. Fractional Atomic Coordinates (×10^4^) and Equivalent Isotropic Displacement Parameters (Å^2^ × 10^3^) for **1**. Table S3. Anisotropic Displacement Parameters (Å^2^ × 10^3^) for **1**. The Anisotropic displacement factor exponent takes the form: −2π^2^[h^2^a*^2^U_11_+2hka*b*U_12_+…]. Table S4. Bond Lengths for **1**. Table S5. Bond Angles for **1**. Table S6. Torsion Angles for **1**. Table S7. Hydrogen Atom Coordinates (Å × 10^4^) and Isotropic Displacement Parameters (Å^2^ × 10^3^) for **1**. Table S8. Crystal data and structure refinement for **6**. Table S9. Fractional Atomic Coordinates (×10^4^) and Equivalent Isotropic Displacement Parameters (Å^2^ × 10^3^) for **6**. U_eq_ is defined as 1/3 of the trace of the orthogonalised U_IJ_ tensor. Table S10. Anisotropic Displacement Parameters (Å^2^ × 10^3^) for **6**. The Anisotropic displacement factor exponent takes the form: −2π^2^[h^2^a*^2^U_11_+2hka*b*U_12_+…]. Table S11. Bond Lengths for **6**. Table S12. Bond Angles for **6**. Table S13. Torsion Angles for **6**. Table S14. Hydrogen Atom Coordinates (Å × 10^4^) and Isotropic Displacement Parameters (Å^2^ × 10^3^) for **6**. Table S15. Cartesian Coordinates for Complex **1**. Table S16. Cartesian Coordinates for Complex **2**. Table S17. Cartesian Coordinates for Complex **3**. Table S18. Cartesian Coordinates for Complex **4**.
