# Supplementary material for: Use of Novel Homochiral Thioureas Camphor Derived as Asymmetric Organocatalysts in the Stereoselective Formation of Glycosidic Bonds
Source: Molecules. 2024 Feb 9;29(4):811. doi: 10.3390/molecules29040811 (PMC10893146; doi:10.3390/molecules29040811)
Supplement: Supplementary file 1 [file molecules-29-00811-s001.zip › molecules-2850428-supplementary.pdf]

Supporting Information

Mildred López [1], Gabriela Huelgas [1], Mario Sánchez [2], Adalid Armenta [2], Angel Mendoza [3], José Daniel Lozada-Ramírez [1] and Cecilia Anaya de Parrodi [1,*]

[1] Departamento de Ciencias Químico-Biológicas, Universidad de las Américas Puebla, Puebla 72810, Mexico; mildred.lopezvz@udlap.mx (M.L.); gabriela.huelgas@udlap.mx (G.H.); jose.lozada@udlap.mx (J.D.L.-R.)
[2] Centro de Investigación en Materiales Avanzados S.C., Alianza Norte 202, PIIT, Apodaca 66628, Mexico; mario.sanchez@cimav.edu.mx (M.S.); adalid.torres@cimav.edu.mx (A.A.)
[3] Centro de Química, Instituto de Ciencias, Benemérita Universidad Autónoma de Puebla, Puebla 72570, Mexico; angel.mendoza@correo.buap.mx
* Correspondence: cecilia.anaya@udlap.mx

Table of Contents

Experimental section S1
Part 1. General information S1
Part 2. General synthesis of homochiral thioureas S2
Part 3. Characterization Data of Compounds 1 – 6. .... S3
Spectral data for thioureas 1 – 6. .... S6
X-ray diffraction data for thioureas 1 and 6 .... S18
Cartesian Coordinates for Complex 1 .... S34

Experimental section

Part 1. General information

All chemicals were obtained from commercial suppliers and used without purification. Liquid chemicals, solutions, or solvents were added using a syringe (mL) or a micropipette (µL). Reactions were followed by thin-layer chromatography using silica gel 60 F254 plates supported in aluminum (Merck, Telos) and were revealed by UV light (254 nm), using phosphomolybdic acid in ethanol (0.25 g/mL), or with iodide vapors. The products obtained were concentrated using Rotavapor Büchi R-300 (bath temperatures above 40 °C) and reduced pressure depending on the solvent used.

^1H and ^{13}C NMR spectra were obtained in $CDCl_3$ using a Bruker AscendTM (400 MHz) spectrometer with tetramethylsilane (TMS) as a reference at 0 ppm. Optical rotation recordings were measured using an Autopol III (Rudolph Research Analytical) polarimeter at 20 °C and 589 nm, using $CHCl_3$ as the blank and solvent, and concentrations were calculated in g/mL. Infrared (IR) spectra were obtained using an FTIR Cary 630 spectrophotometer (Agilent Technologies).

Single-crystal XRD analysis. A single crystal of each compound was mounted on a loop plastic fiber. Diffraction analyses were carried out on an Oxford Diffraction Gemini "Atlas" diffractometer, equipped with a charge-coupled device area detector, sealed X-ray tube ($\lambda MoK\alpha$ = 0.71073 Å), and a graphite monochromator. The CrysAlis PRO and CrysA-lis RED software packages were used for data collection and integration. The collected data were corrected for absorbance using an analytical numerical correction with a multifaceted crystal model. Structure solution and refinement were carried out using Olex2 software. To prepare the material for publication, Mercury 4.0 and Olex2 software were used.

Part 2. General synthesis of homochiral thioureas

To a solution of the chiral aminoalcohol[1] (1*R*,2*S*,3*R*,4*S*)-**14** (1 equiv) in CH_2Cl_2 (10 mL) were added, (*S*)-1-methylbenzyl isothiocyanate, (*R*)-1-methylbenzyl isothiocyanate, (3,5-trifluoromethyl) phenyl isothiocyanate, benzhydryl isothiocyanate, benzyl isothiocy-anate and phenyl isothiocyanate (1.1 equiv)[2] under a nitrogen atmosphere. The mixture was stirred at room temperature for 2 h, then the solvent was eliminated by reduced pressure and the products obtained were purified using a chromatographic column with basic silica (triethylamine/SiO_2 = 2.0% v/w, hexane/ethyl acetate 4:1 v/v) to obtain thioureas **1 – 6**.

Part 3. Characterization Data of Compounds 1 – 6.

1-((1R,2S,3R,4S)-2-Hydroxy-1,7,7-trimethylbicyclo[2.2.1]heptan-3-yl)-3-((S)-1-phenylethyl)thiourea-**1**

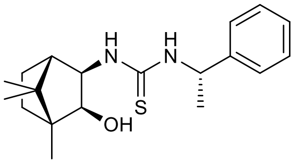

(1R,2S,3R,4S,12S)-**1**

Crystals (79% yield), m.p. 111 – 113 °C. $[\alpha]_D$ = +90.08 (c = 0.086, $CHCl_3$). ^1H NMR (400 MHz, $CDCl_3$, δ): 0.63 (s, 3H), 0.69 (s, 3 H), 0.87 (s, 3 H), 1.01-1.15 (m, 2 H), 1.44 (td, J = 2.8, 12.0 Hz, 1 H), 1.5 (d, J = 8 Hz, 3 H), 1.60-1.69 (m, 2 H), 2.55 (b, 1 H), 3.80-3.82 (m, 1 H), 3.95 (t, J = 8.0, 4.0 Hz, 1 H), 4.64 (b, 1 H), 6.04 (b, 1 H), 6.39 (b, 1 H), 7.26-7.38 (m, 5 H). ^{13}C NMR (100 MHz, $CDCl_3$, δ): 11.2, 20.5, 21.3, 24.2, 25.9, 33.2, 46.8, 49.1, 50.4, 54.1, 63.0, 80.1, 125.7, 128.2, 129.3, 142.0, 180.0 IR-FT: 3334, 3256, 2954, 2873, 2290, 2169, 1700, 1522, 1449, 1407, 1378, 1347, 1318, 1242, 1215, 1123, 1147, 1089, 1054, 963, 814, 748, 696, 664, 607 cm^{-1}. HRMS (FAB^+) m/z calcd. for [$C_{19}H_{29}ON_2S$]: 333.1917, found 333.1972. Recrystallized from hexanes/CH_2Cl_2 (5:1), 0.526 × 0.246 × 0.195 mm^3, $C_{19}H_{28}N_2OS$ (M = 332.49 g/mol): monoclinic, $P2_1$ (no.4) a = 7.6886(3) Å, b = 27.9174(10) Å, c = 8.9652(4) Å, β = 101.610(4)°, V = 1884.97(13) $Å^3$, Z = 4, T = 293(2) K , μ = 0.178 mm^{-1}, ρ_{calcd} = 1.172 g/cm^3, 25311 reflections measured (5.838° ≤ 2Θ ≤ 61.012), [Rint = 0.0581, R_{sigma} = 0.0727]. F(000) = 720.0. Final R_1 was 0.0821 (I > 2σ(I)) and wR_2 was 0.1392 for all data. CCDC deposition number: 2302046.

1-((1R,2S,3R,4S)-2-Hydroxy-1,7,7-trimethylbicyclo[2.2.1]heptan-3-yl)-3-((R)-1-phenylethyl)thiourea-**2**

White solid (90% yield), m.p. 65 - 67 °C. $[\alpha]_D$ = +106.37 (c = 0.014, CHCl3). ^1H NMR (400 MHz, $CDCl_3$, δ): 0.60 (s, 3H), 0.71 (s, 3H), 0.83 (s, 3H), 0.99-1.06 (m, 1 H), 1.10-1.17 (m, 1 H), 1.44 (td, J = 4.0, 12.0 Hz, 1H), 1.49 (d, J = 4 Hz, 3 H), 1.64-1.72 (m, 1 H), 1.77 (d, J = 4 Hz, 1 H), 2.31 (b, 1 H), 3.73 (d, J = 8.0 Hz, 1H), 3.90 (m, 1H), 4.65 (b, 1H), 6.05 (b, 1H), 6.46 (b, 1H), 7.25 - 7.38 (m, 5 H). ^{13}C NMR (100 MHz, CDCl3, δ): 11.2, 20.1, 21.3, 24.5, 26.1, 33.2, 47.1, 49.0, 50.4, 54.2, 63.5, 80.4, 125.7, 128.2, 129.3, 141.8, 180.5. IR-FT: 3275, 3048, 2951, 2935, 2100, 1890, 1704, 1520, 1341, 1239, 1052, 924, 758. HRMS (FAB+) m/z calcd. for [$C_{19}H_{29}ON_2S$]: 333.1922 found 333.1978.

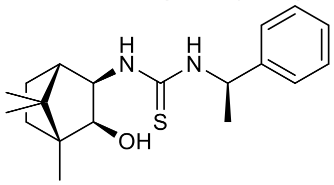

(1R,2S,3R,4S,12R)-**2**

3-[3,5-Bis(trifluoromethyl)phenyl]-1-[(1*R*,2*S*,3*R*,4*S*)-2-hydroxy-1,7,7-trimethylbicyclo[2.2.1]heptan-3-yl]thiourea-**3**

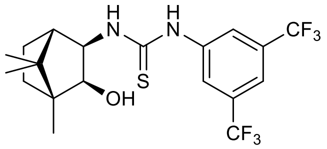

(1*R*,2*S*,3*R*,4*S*)-**3**

White crystals (64% yield), m.p. 184 – 186 °C. $[\alpha]_D$ = +77.12 (c = 0.024, CHCl3), 1H NMR (400 MHz, CDCl3, δ): 0.73 (s, 3H), 0.84 (s, 3H), 0.88 (s, 3H), 0.99-1.03 (m, 1H), 1.13-1.18 (m, 1H), 1.46 (td, J = 4.0, 12.0 Hz, 1H), 1.64-1.69 (m, 1H), 1.94 (d, J = 4.0 Hz, 1H), 2.46 (b, 1H), 3.80 (dd, J = 4.0, 8.0 Hz, 1H), 4.11 (b, 1H), 7.00 (b, 1H), 7.61 (s, 1H), 7.69 (s, 2H), 8.84 (b, 1H). 13C NMR (100 MHz, CDCl3, δ): 11.1, 20.9, 21.3, 25.9, 33.0, 46.7, 49.3, 50.2, 62.6, 79.8, 119.3, 122.7 (q, ^{1}JCF = 271 Hz, CF3), 123.7, 133.2, 179.2. IR-FT: 3155, 3031, 2960, 1622, 1552, 1505, 1467, 1375, 1274, 1170, 1122, 1053, 963, 887, 846, 792, 707, 680 cm^{-1}. HRMS (FAB+) m/z calcd. for [$C_{19}H_{23}ON_2F_6S$]: 441.1357, found 441.1339.

1-Benzhydryl-3-((1R,2*S*,3*R*,4*S*)-2-hydroxy-1,7,7-trimethylbicyclo[2.2.1]heptan-3-yl)thiourea-**4**

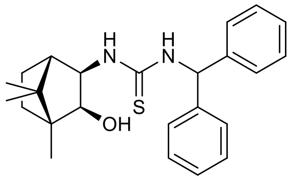

(1*R*,2*S*,3*R*,4*S*)-**4**

White crystals (79% yield), m.p. 152 - 154°C. [α]D = +70.27 (c = 0.023, $CHCl_3$). ^{1}H NMR (400 MHz, $CDCl_3$, δ): 0.50 (s, 3 H), 0.63 (s, 3H), 0.78 (s, 3H), 0.89-1.10 (m, 2H), 1.19 (s, 1H) , 1.34-1.41 (m, 1H), 1.56-1.63 (m, 1H), 2.38 (b, 1H), 3.72 (d, J = 7.2, 1 H), 3.90 (b, 1H), 5.66 (b, 1H), 6.09 (b, 1H), 6.44 (b, 1H), 7.19-7.29 (m, 10H). ^{13}C NMR (100 MHz, $CDCl_3$, δ): 11.4, 20.3, 21.4, 26.0, 33.2, 46.9, 49.1, 50.3, 62.5, 63.1, 79.9, 127.3, 127.4, 128.2, 129.1, 140.0, 180.0 IR-FT: 3675, 3263, 2952, 2928, 2079, 1873, 1737, 1523, 1503, 1305, 1217, 1055, 960, 803, 758, 740, 697 cm^{-1}. HRMS (FAB+) m/z calcd. for [C_{24}H31ON_2S]: 395.2079 found 395.2220.

1-Benzyl-3-((1R,2S,3R,4S)-2-hydroxy-1,7,7-trimethylbicyclo[2.2.1]heptan-3-yl)thiourea-**5**

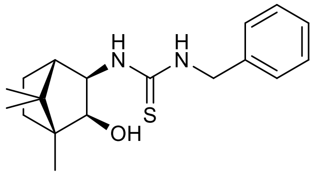

(1*R*,2*S*,3*R*,4*S*)-**5**

White solid (74% yield), m.p. 138-139 °C. $[\alpha]_D$ = +104.28 (c = 0.007, CHCl3). ^{1}H NMR (400 MHz, CDCl3, δ): 0.68 (s, 3 H), 0.80 (s, 3 H), 0.82 (s, 3 H), 0.94-1.09 (m, 2 H), 1.40 (td, J = 4.0, 12.0 Hz, 1 H), 1.57-1.66 (m, 1 H), 1.75 (d, J = 4.0 Hz, 1 H), 2.96 (b, 1H), 3.74 (d, J = 8.0 Hz, 1H), 3.81 (b, 1H), 4.47 (b, 2 H), 6.42 (b, 2 H), 7.19-7.27 (m, 5 H). ^{13}C NMR (100 MHz, CDCl3, δ): 11.3, 20.8, 21.4, 26.0, 33.2, 47.0, 48.1, 49.0, 50.2, 62.4, 79.9, 127.4, 128.0, 129.0, 136.7, 180.9. IR-FT: 3256, 2949, 2872, 2730, 1528, 1455, 1343, 1307, 1269, 1238, 1123, 1192, 1092, 1052, 961, 856, 797, 731, 694, 645 cm-1. HRMS (FAB+) m/z calcd. for [$C_{18}H_{27}ON_2S$]: 319.1766, found 319.1836.

1-((1*R*,2*S*,3*R*,4*S*)-2-Hydroxy-1,7,7-trimethylbicyclo[2.2.1]heptan-3-yl)-3-phenylthiourea **6**

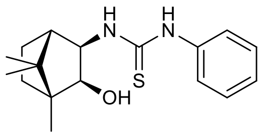

(1*R*,2*S*,3*R*,4*S*)-**6**

White solid (93% yield), m.p. 171-173 °C. $[\alpha]_D$ = +106.04 (c = 0.008, CHCl3), ^{1}H NMR (400 MHz, CDCl3, δ): 0.77 (s, 3 H), 0.90 (s, 6H), 1.06-1.13 (m, 1H), 1.18-1.26 (m, 1H), 1.50 (td, J = 4.0, 12.0 Hz, 1H), 1.70-1.74 (m, 1H), 1.86 (d, J = 8.0 Hz, 1H), 2.62 (b, 1H), 3.90 (d, J = 8.0 Hz, 1H), 4.14 (t, J = 4.0, 8.0 Hz, 1H), 6.64 (d, J = 8.0 Hz, 1H), 7.20-7.22 (m, 2 H), 7.27-7.31 (m, 1H), 7.39-7.43 (m, 2 H), 7.98 (b, 1 H) ^{13}C NMR (100 MHz, CDCl3, δ): 11.3, 20.9, 21.3, 26.1, 33.2, 46.9, 49.3, 50.3, 63.0, 80.3, 125.2, 127.3, 130.0, 136.1, 179.6. IR-FT: 3431, 3392, 3378, 3218, 3105, 2921, 2338, 2099, 1670, 1614, 1535, 1505, 1391, 1244,1053, 923, 839, 788, 693 cm-1. HRMS (FAB+) m/z calcd. for $[C_{17}H_{25}ON_2S]$: 305.1609, found 305.1726. Recrystallized from hexanes/CH_2Cl_2 (5:1), 0.52 × 0.33 × 0.21 mm^3, $C_{17}H_{24}N_2OS$ (*M* = 304.44 g/mol): monoclinic, $P2_1$ (no. 4), *a* = 7.6667(2) Å, *b* = 11.9881(2) Å, *c* = 18.6749(4) Å, *β* = 100.548(2)°, *V* = 1687.39(6) Å^3, *Z* = 4, T = 293(2) K, μ = 0.193 mm^{-1}, *ρcalcd*=1.198 g/cm^3, 54301 reflections measured (6.208° ≤ 2Θ ≤ 61.012°), 10257 unique [R_{int} = 0.0384, R_{sigma} = 0.0291]. F(000) = 656.0. Final R_1 was 0.0436 ((I > 2σ(I)), wR_2 was 0.1248 for all data. CCDC deposition number: 2302045

Spectral data for thioureas 1 – 6.

Figure S1. ^{1}H NMR of 1-((1*R*,2*S*,3*R*,4*S*)-2-Hydroxy-1,7,7-trimethylbicyclo[2.2.1]heptan-3-yl)-3-((*S*)-1-phenylethyl)thiourea-**1**

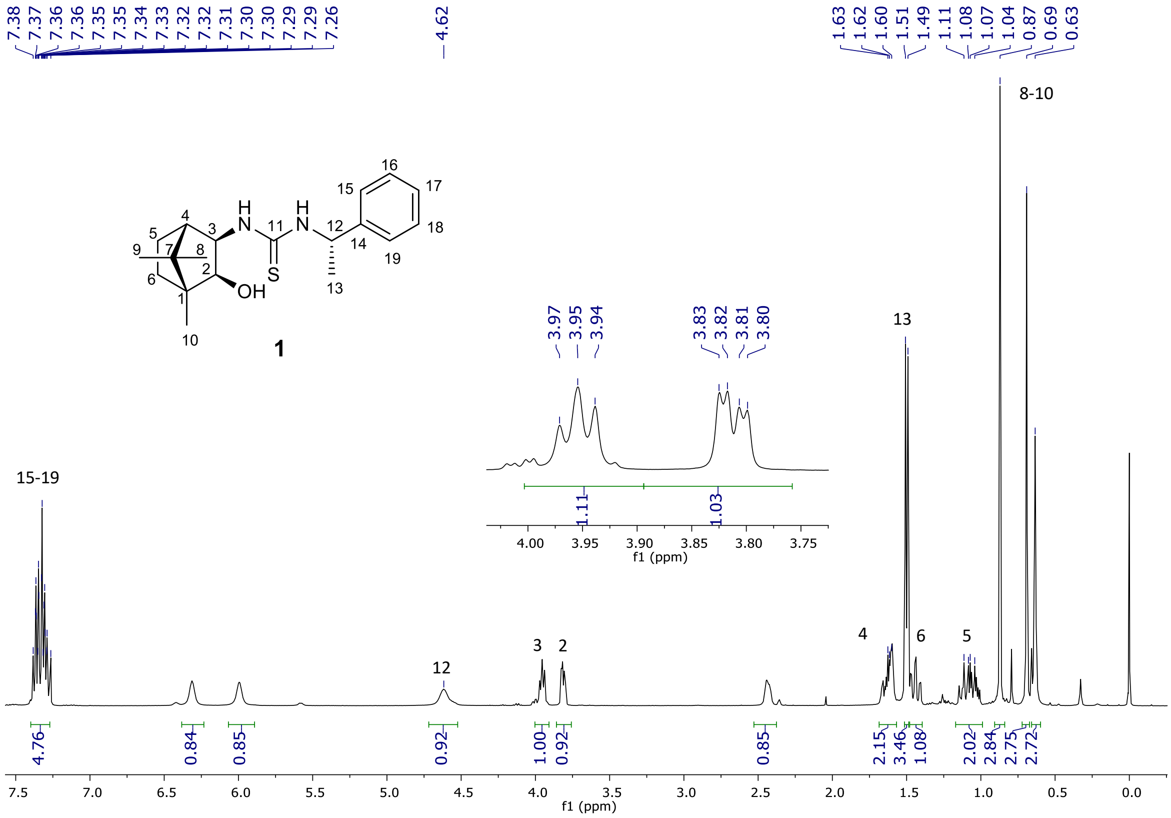

Figure S2. ^{13}C NMR of 1-((1*R*,2*S*,3*R*,4*S*)-2-Hydroxy-1,7,7-trimethylbicyclo[2.2.1]heptan-3-yl)-3-((*S*)-1-phenylethyl)thiourea-**1**

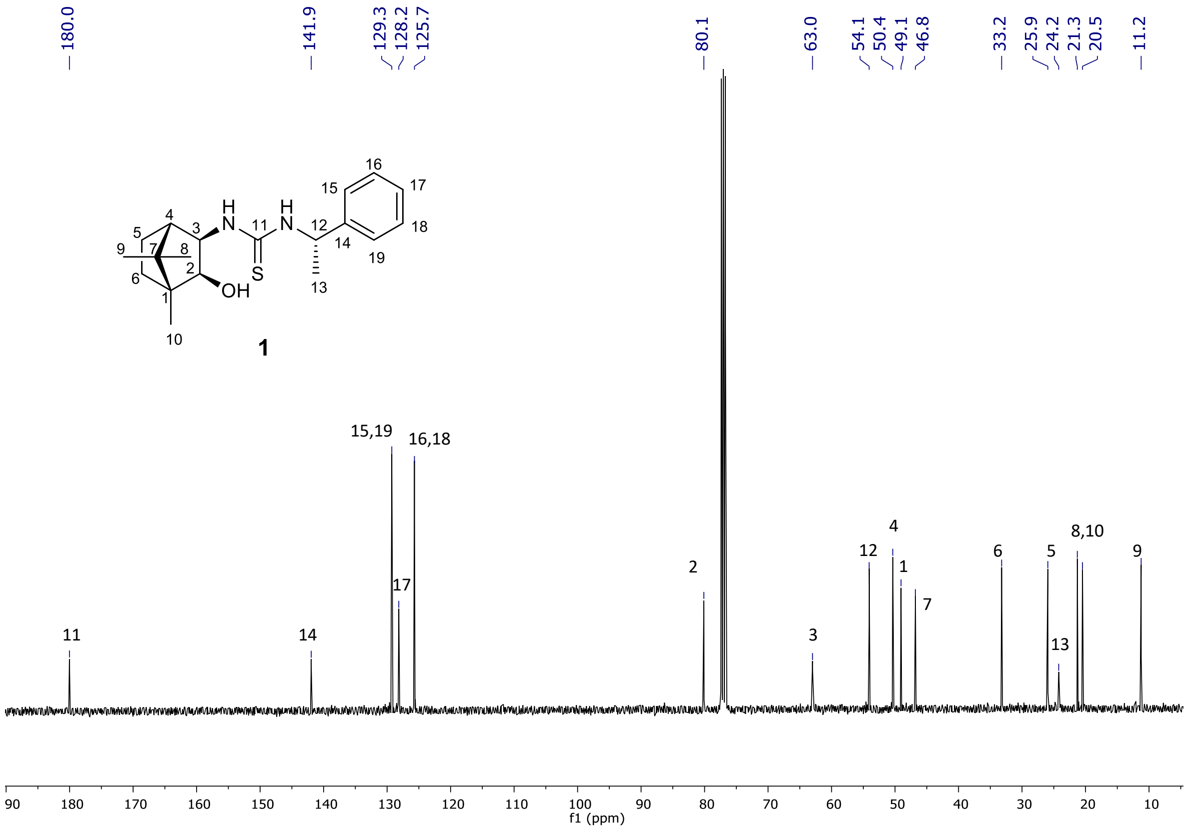

Figure S3. HRMS of 1-((1*R*,2*S*,3*R*,4*S*)-2-Hydroxy-1,7,7-trimethylbicyclo[2.2.1]heptan-3-yl)-3-((*S*)-1-phenylethyl)thiourea-**1**

```
[ Elemental Composition ]
Data  : 030-LEM-HR                    Date : 23-Jun-2023 07:35

Sample: (S)-cat.3             Operator name M.en ITA Victoria Labastida G.   I
Note : UDLAP Mildred López  Centro de Investigaciones Químicas UAEM

Inlet : Direct                        Ion Mode : FAB+
RT : 0.53 min                         Scan#: 11+153+(7,13)

Elements : C 40/0, H 49/0, O 1/0, N 2/0, S 1/0
Mass Tolerance      : 1000ppm, 3mmu if m/z < 3, 5mmu if m/z > 5

Unsaturation (U.S.) : -0.5 - 10.0

Observed m/z Int%   Err[ppm / mmu]    U.S.  Composition
  333.1972   100.0     -8.5 /  -2.8    7.5  C 19 H 29 O N 2 S
```

Figure S4. ^{1}H NMR of 1-((1*R*,2*S*,3*R*,4*S*)-2-Hydroxy-1,7,7-trimethylbicyclo[2.2.1]heptan-3-yl)-3-((*R*)-1-phenylethyl)thiourea-**2**

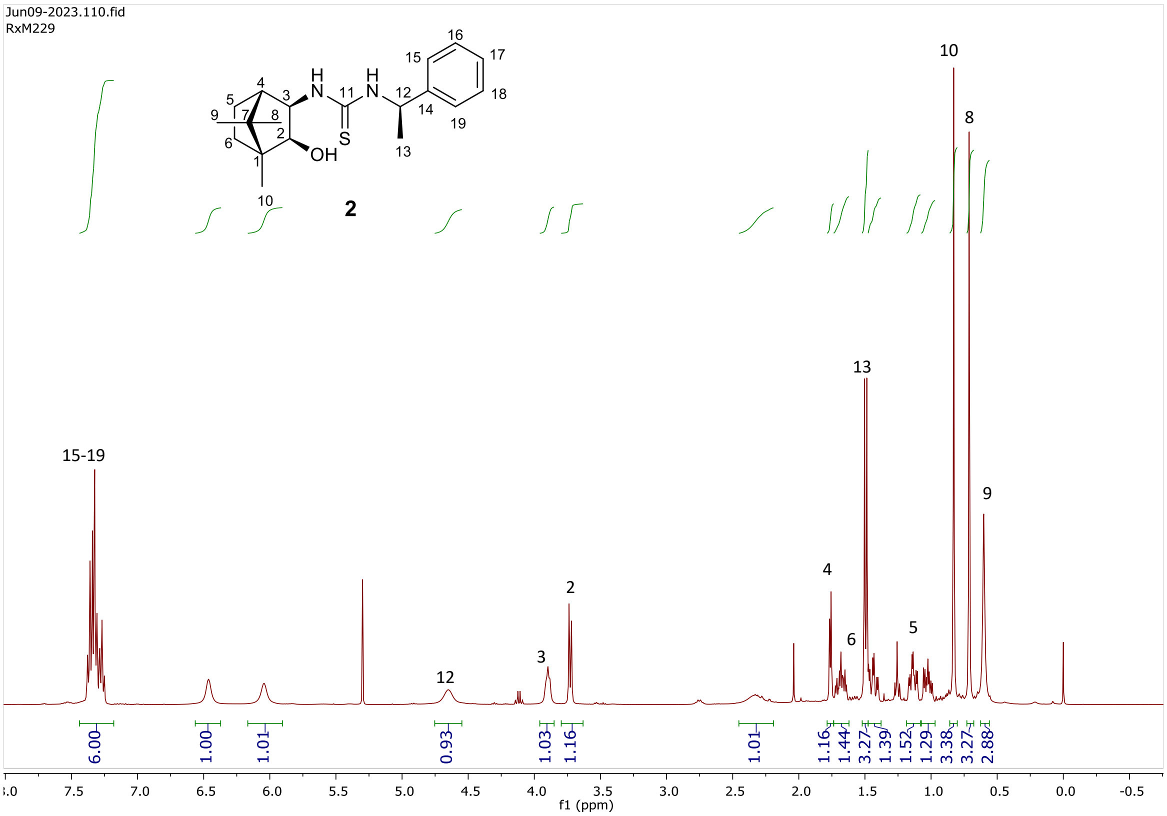

Figure S5. ^{13}C NMR of 1-((1*R*,2*S*,3*R*,4*S*)-2-Hydroxy-1,7,7-trimethylbicyclo[2.2.1]heptan-3-yl)-3-((*R*)-1-phenylethyl)thiourea-**2**

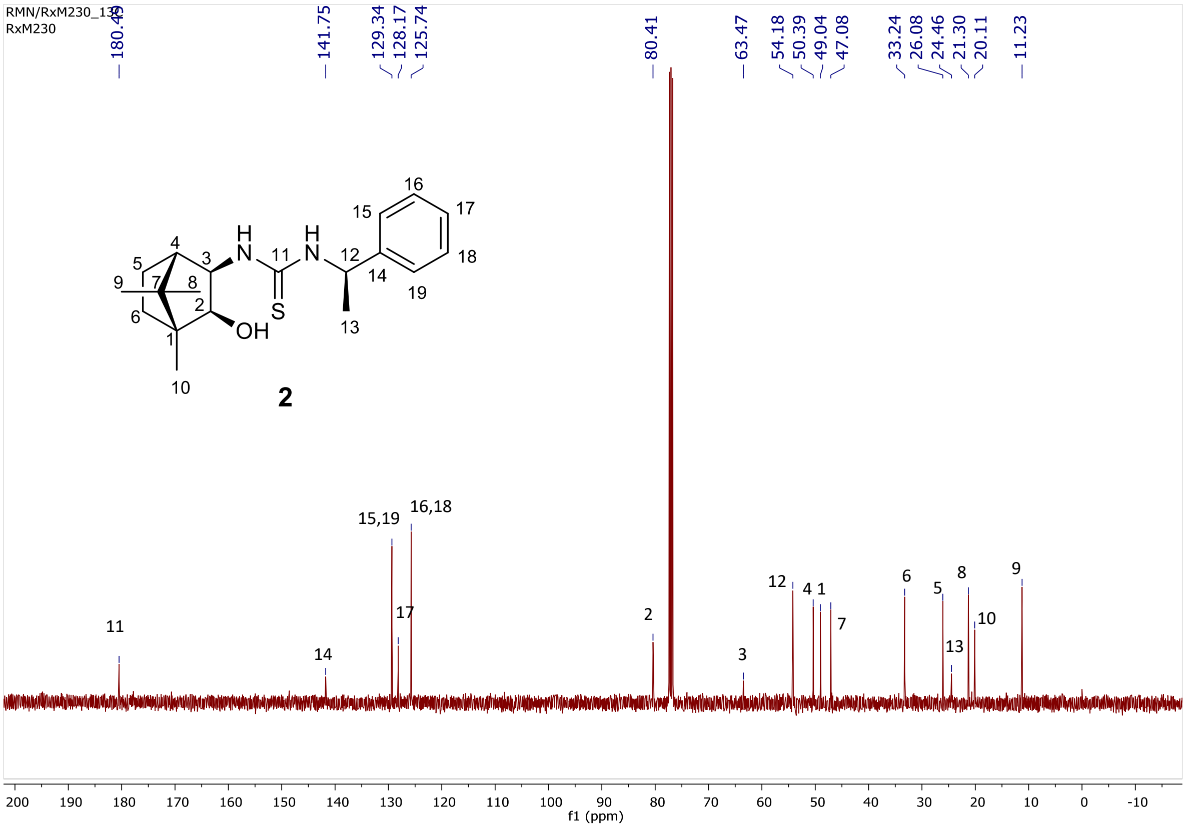

Figure S6. HRMS of 1-((1*R*,2*S*,3*R*,4*S*)-2-Hydroxy-1,7,7-trimethylbicyclo[2.2.1]heptan-3-yl)-3-((*R*)-1-phenylethyl)thiourea-**2**

```
[ Elemental Composition ]
Data  : 034-LEM-HR                    Date : 20-Jul-2023 12:23

Sample: RxM230                  Operator name M.en ITA Victoria Labastida G.
Note : UDLAP/Mildred López  Centro de Investigaciones Químicas UAEM

Inlet : Direct                        Ion Mode : FAB+
RT : 0.06 min                         Scan#: 2+48

Elements : C 40/0, H 49/0, O 1/0, N 2/0, S 1/0
Mass Tolerance      : 1000ppm, 3mmu if m/z < 3, 5mmu if m/z > 5

Unsaturation (U.S.) : -0.5 - 10.0

Observed m/z Int%   Err[ppm / mmu]    U.S.  Composition
  333.1978   100.0     -6.8 /  -2.3    7.5  C 19 H 29 O N 2 S
```

Figure S7. ^{1}H NMR of 3-[3,5-Bis(trifluoromethyl)phenyl]-1-[(1*R*,2*S*,3*R*,4*S*)-2-hydroxy-1,7,7-trimethylbicyclo[2.2.1]heptan-3-yl]thiourea-**3**

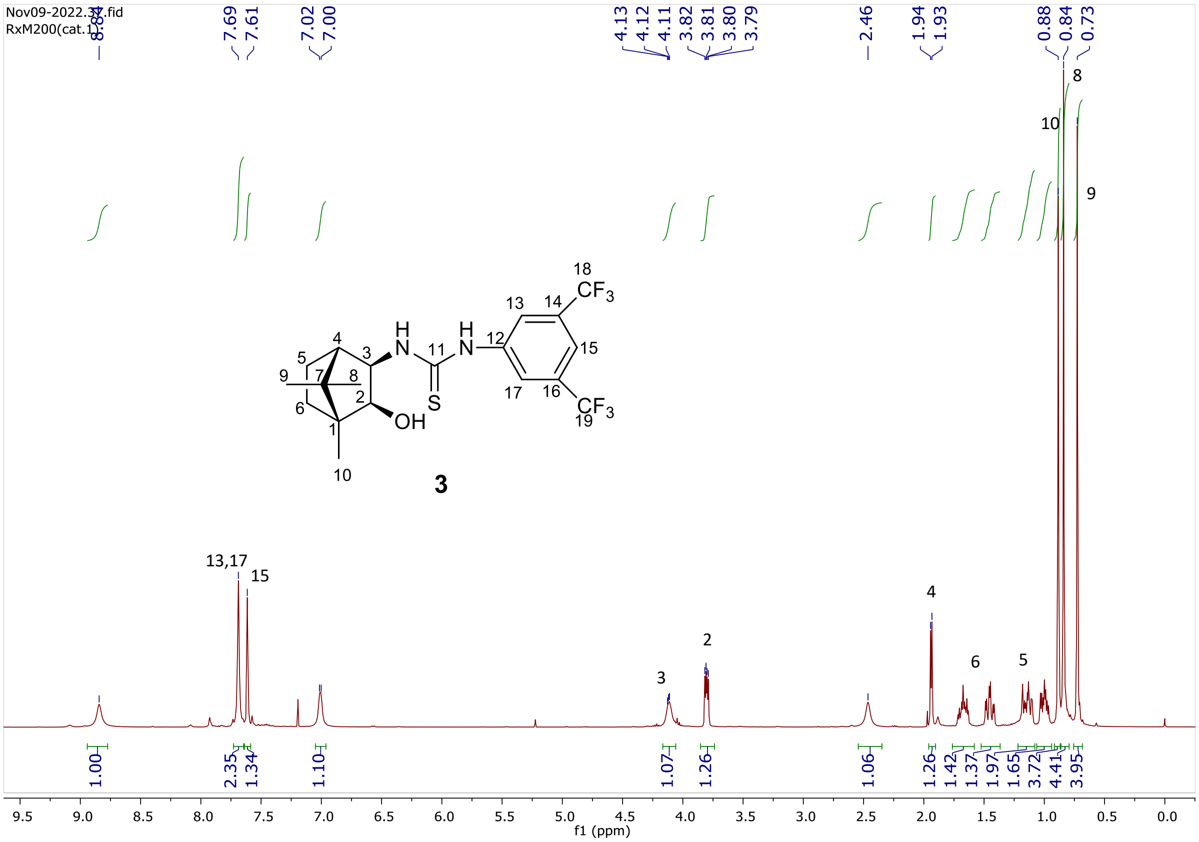

Figure S8. ^{13}C NMR of 3-[3,5-Bis(trifluoromethyl)phenyl]-1-[(1*R*,2*S*,3*R*,4*S*)-2-hydroxy-1,7,7-trimethylbicyclo[2.2.1]heptan-3-yl]thiourea-**3**

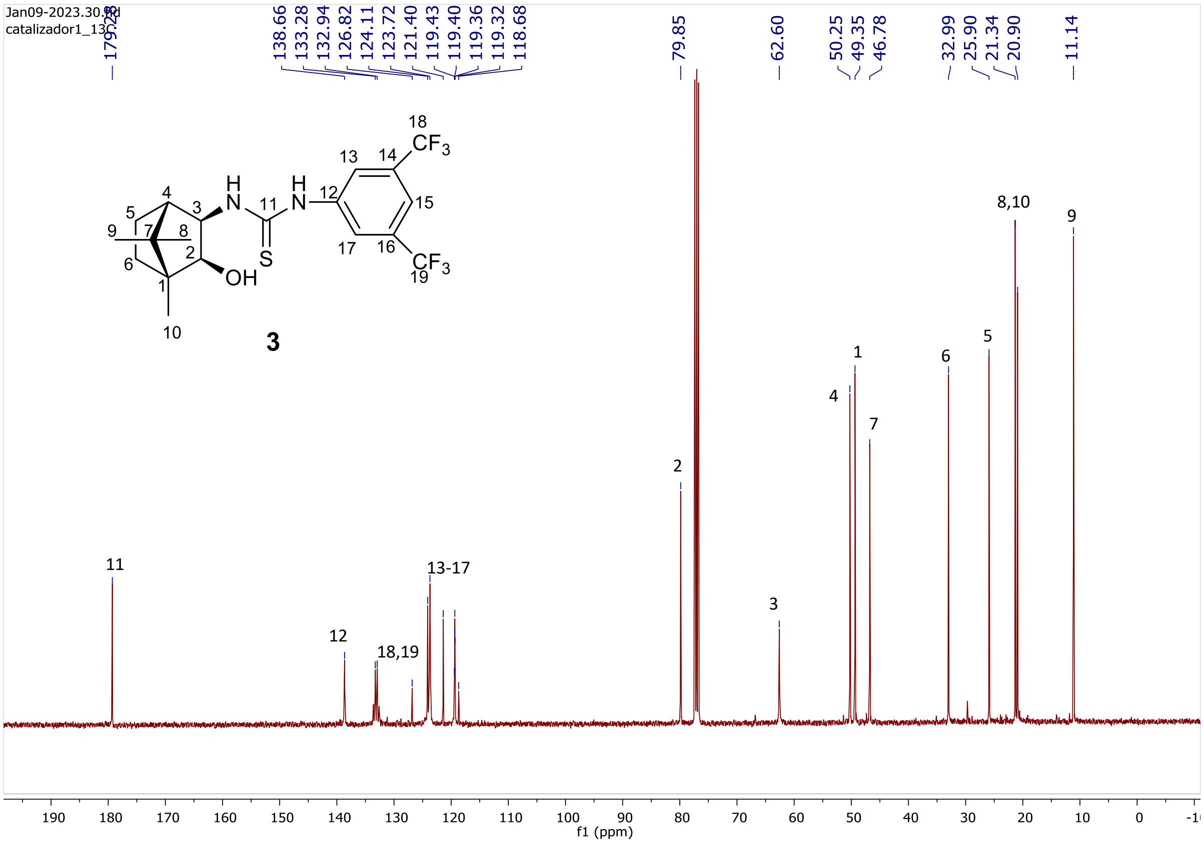

Figure S9. HRMS of 3-[3,5-Bis(trifluoromethyl)phenyl]-1-[(1*R*,2*S*,3*R*,4*S*)-2-hydroxy-1,7,7-trimethylbicyclo[2.2.1]heptan-3-yl]thiourea-**3**

```
[ Elemental Composition ]
Data  : 011LEM-HR_Correccion              Date : 24-Feb-2023 13:05

Sample: RxM77/cat.1           Operator name M.en ITA Victoria Labastida G.  I
Note : UAP/CQB Mildred López Centro de Investigaciones Químicas UAEM

Inlet : Direct                       Ion Mode : FAB+
RT : 0.13 min                        Scan#: 4+110

Elements : C 40/0, H 49/0, O 1/0, N 2/0, F 6/0, S 1/0
Mass Tolerance      : 1000ppm, 3mmu if m/z < 3, 10mmu if m/z > 10

Unsaturation (U.S.) : -0.5 - 10.0

Observed m/z Int%   Err[ppm / mmu]    U.S.  Composition
  441.1339   100.0    -21.8 /  -9.6    7.5  C 19 H 23 O N 2 F 6 S
```

Figure S10. ^{1}H NMR of 1-Benzhydryl-3-((1*R*,2*S*,3*R*,4*S*)-2-hydroxy-1,7,7-trimethylbicyclo[2.2.1]heptan-3-yl)thiourea-**4**

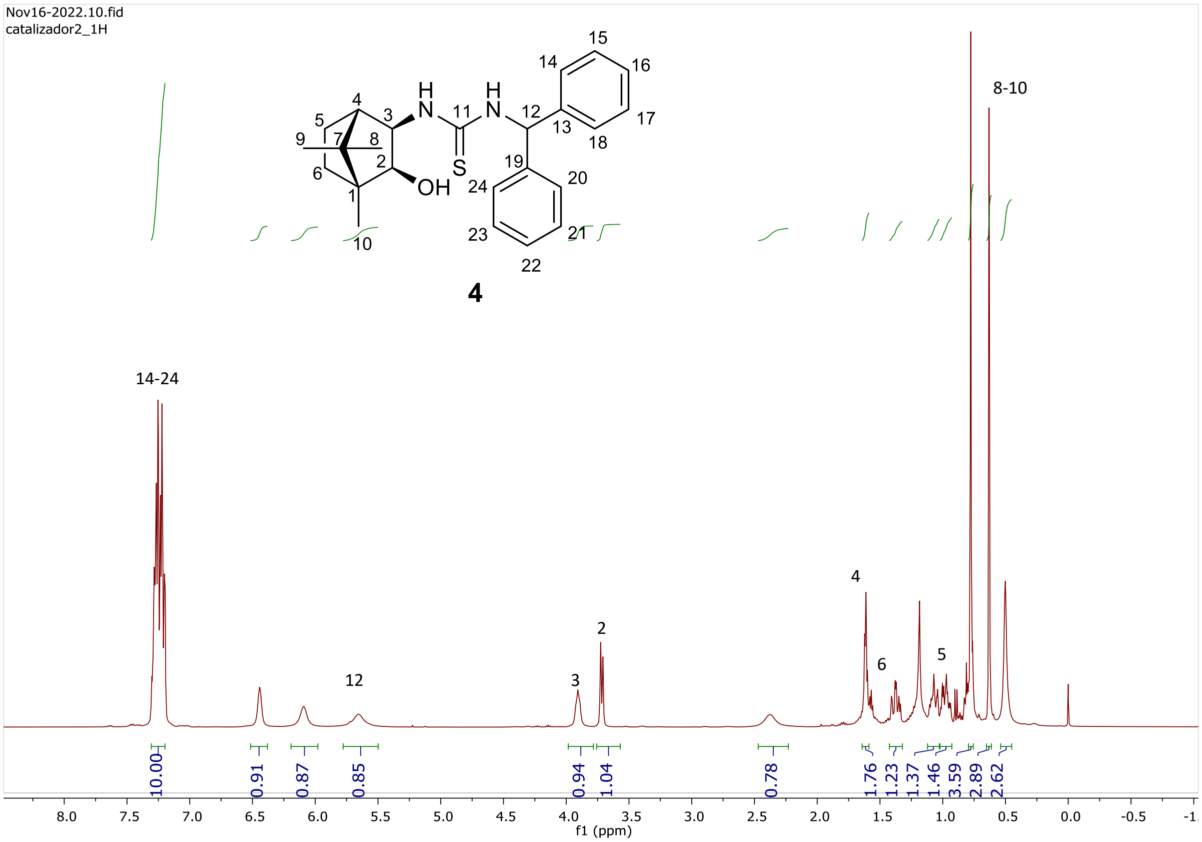

Figure S11. ^{13}C NMR of 1-Benzhydryl-3-((1*R*,2*S*,3*R*,4*S*)-2-hydroxy-1,7,7-trimethylbicyclo[2.2.1]heptan-3-yl)thiourea-**4**

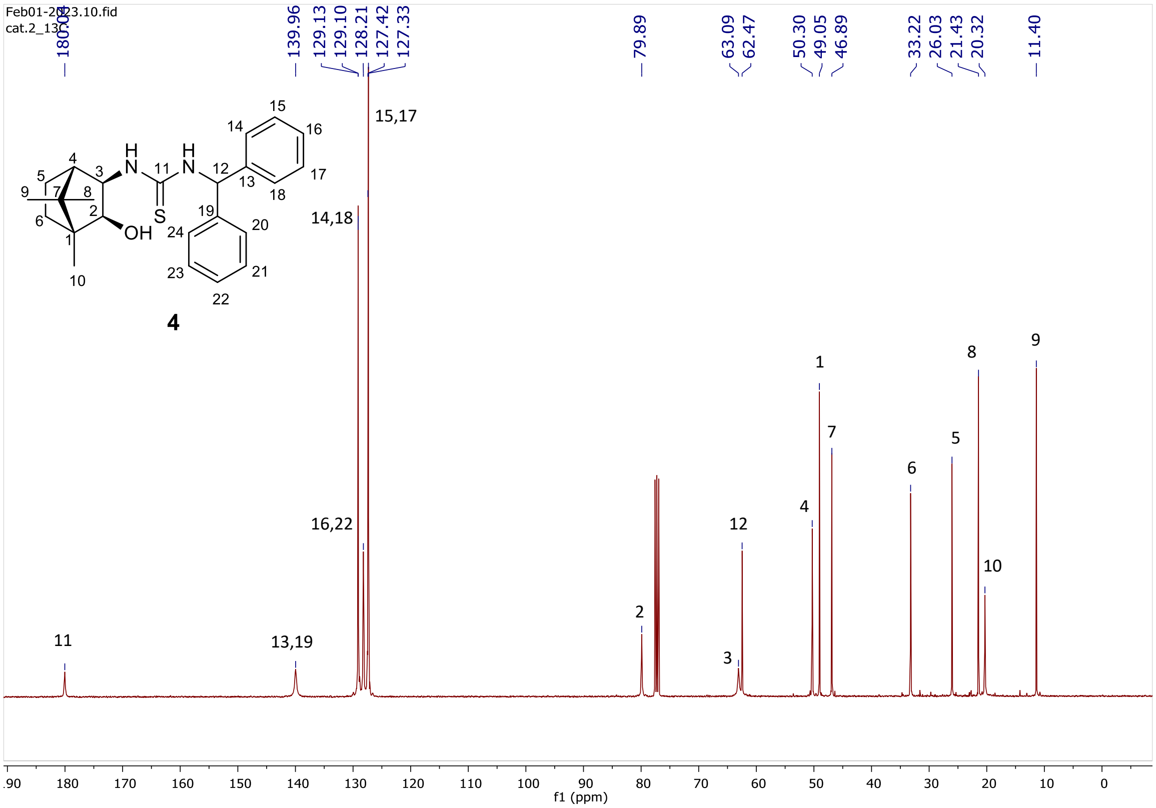

Figure S12. HRMS of 1-Benzhydryl-3-((1*R*,2*S*,3*R*,4*S*)-2-hydroxy-1,7,7-trimethylbicyclo[2.2.1]heptan-3-yl)thiourea-**4**

```
[ Elemental Composition ]
Data  : 012LEM-HR_Correccion                    Date : 24-Feb-2023 13:42

Sample: RxM82/cat.2            Operator name M.en ITA Victoria Labastida G.  I
Nota : UAP/CQB Mildred López Centro de Investigaciones Químicas UAEM

Inlet : Direct                        Ion Mode : FAB+
RT : 0.05 min                         Scan#: 2+48

Elements : C 40/0, H 49/0, O 1/0, N 3/0, S 1/0
Mass Tolerance      : 1000ppm, 3mmu if m/z < 3, 7mmu if m/z > 7

Unsaturation (U.S.) : 11.5 - 11.5

Observed m/z Int%   Err[ppm / mmu]    U.S.  Composition
  395.2220   100.0    +16.0 /  +6.3   11.5  C 24 H 31 O N 2 S
```

Figure S13. ^{1}H NMR of 1-Benzyl-3-((1*R*,2*S*,3*R*,4*S*)-2-hydroxy-1,7,7-trimethylbicyclo[2.2.1]heptan-3-yl)thiourea-**5**

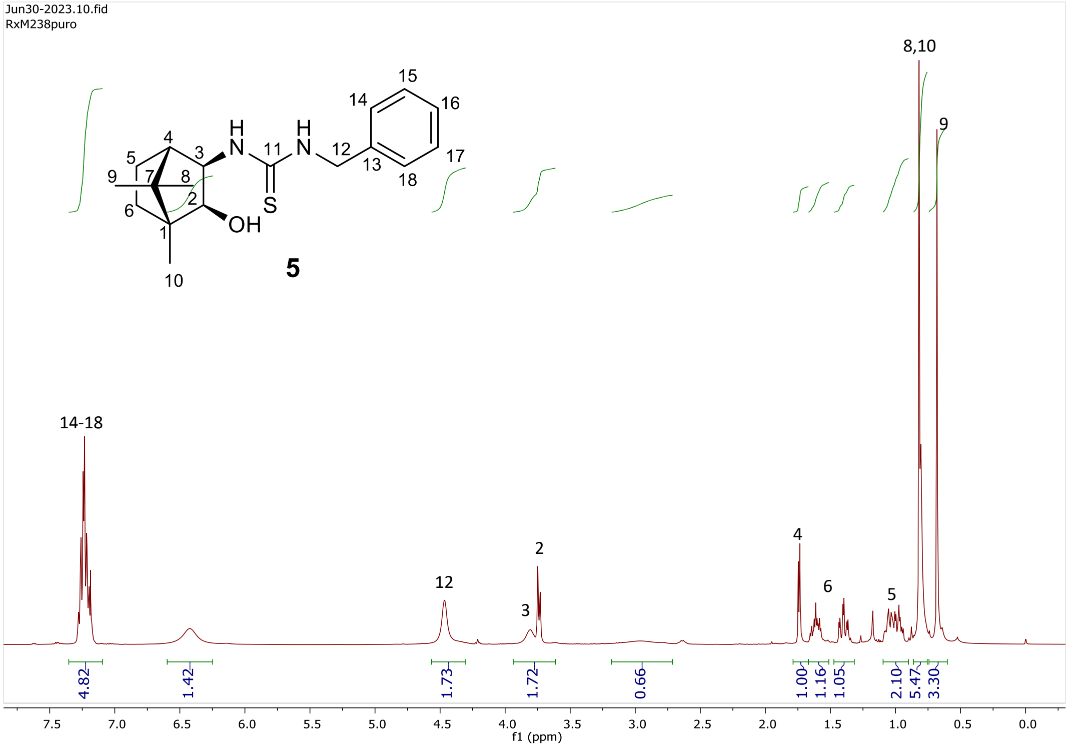

Figure S14. ^{13}C NMR of 1-Benzyl-3-((1*R*,2*S*,3*R*,4*S*)-2-hydroxy-1,7,7-trimethylbicyclo[2.2.1]heptan-3-yl)thiourea-**5**

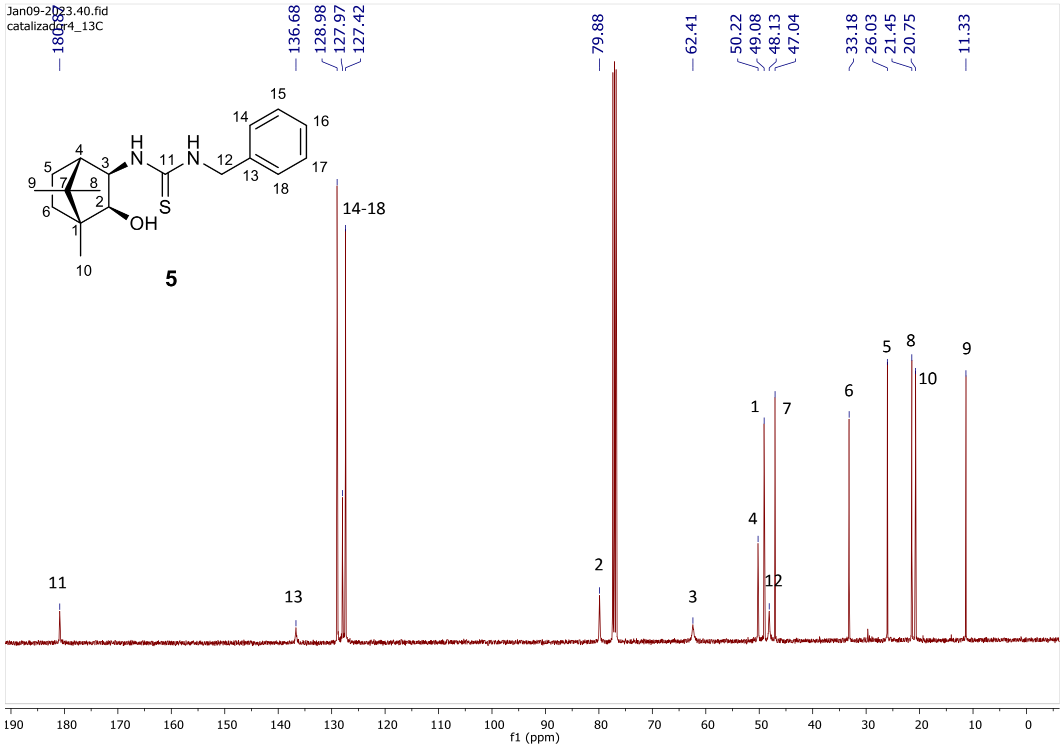

Figure S15. HRMS of 1-Benzyl-3-((1*R*,2*S*,3*R*,4*S*)-2-hydroxy-1,7,7-trimethylbicyclo[2.2.1]heptan-3-yl)thiourea-**5**

```
[ Elemental Composition ]
Data  : 014LEM-HR_Correcion                  Date : 27-Feb-2023 09:38

Sample: RxM103/cat.4            Operator name M.en ITA Victoria Labastida G.
Note  : ULA/CQB Mildred López   Centro de Investigaciones Químicas UAEM

Inlet : Direct                        Ion Mode : FAB+
RT : 0.07 min                         Scan#: 3+55+52

Elements : C 40/0, H 49/0, O 1/0, N 3/0, S 1/0
Mass Tolerance      : 1000ppm, 3mmu if m/z < 3, 5mmu if m/z > 5

Unsaturation (U.S.) : -0.5 - 10.0

Observed m/z Int%   Err[ppm / mmu]    U.S.  Composition
  319.1836   100.0     -2.5 /  -0.8    7.5  C 18 H 27 O N 2 S
```

Figure S16. ^{1}H NMR of 1-((1*R*,2*S*,3*R*,4*S*)-2-Hydroxy-1,7,7-trimethylbicyclo[2.2.1]heptan-3-yl)-3-phenylthiourea **6**

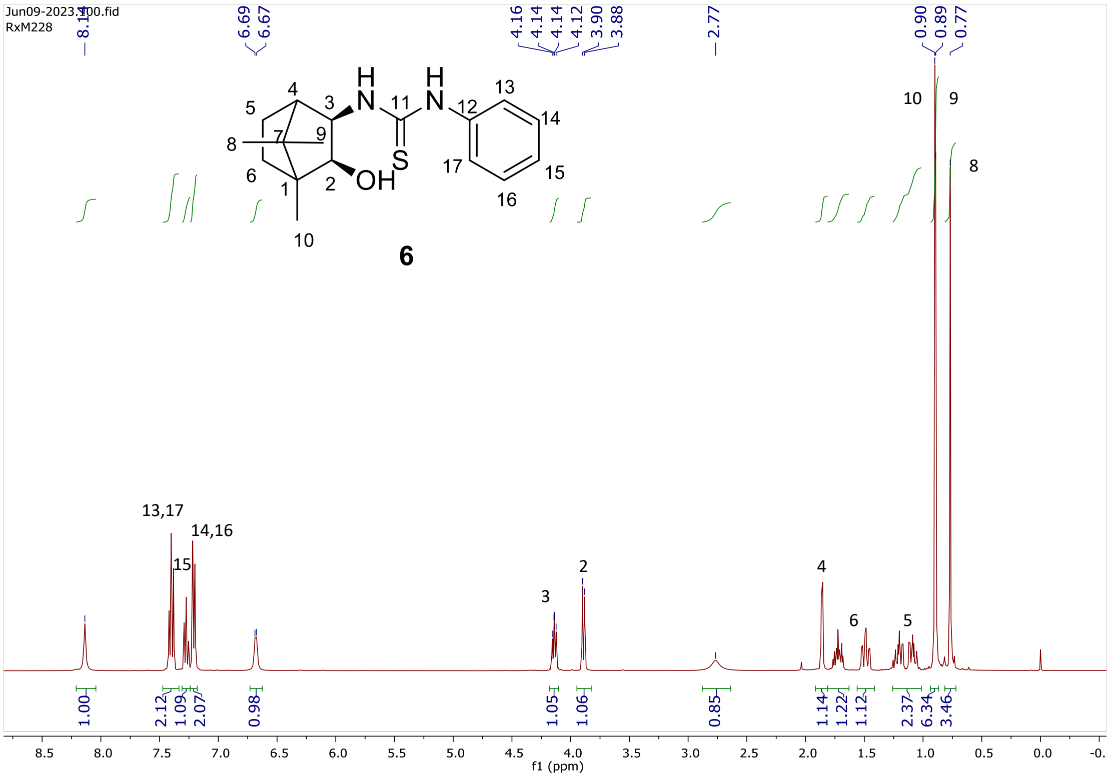

Figure S17. ^{13}C NMR of 1-((1*R*,2*S*,3*R*,4*S*)-2-Hydroxy-1,7,7-trimethylbicyclo[2.2.1]heptan-3-yl)-3-phenylthiourea **6**

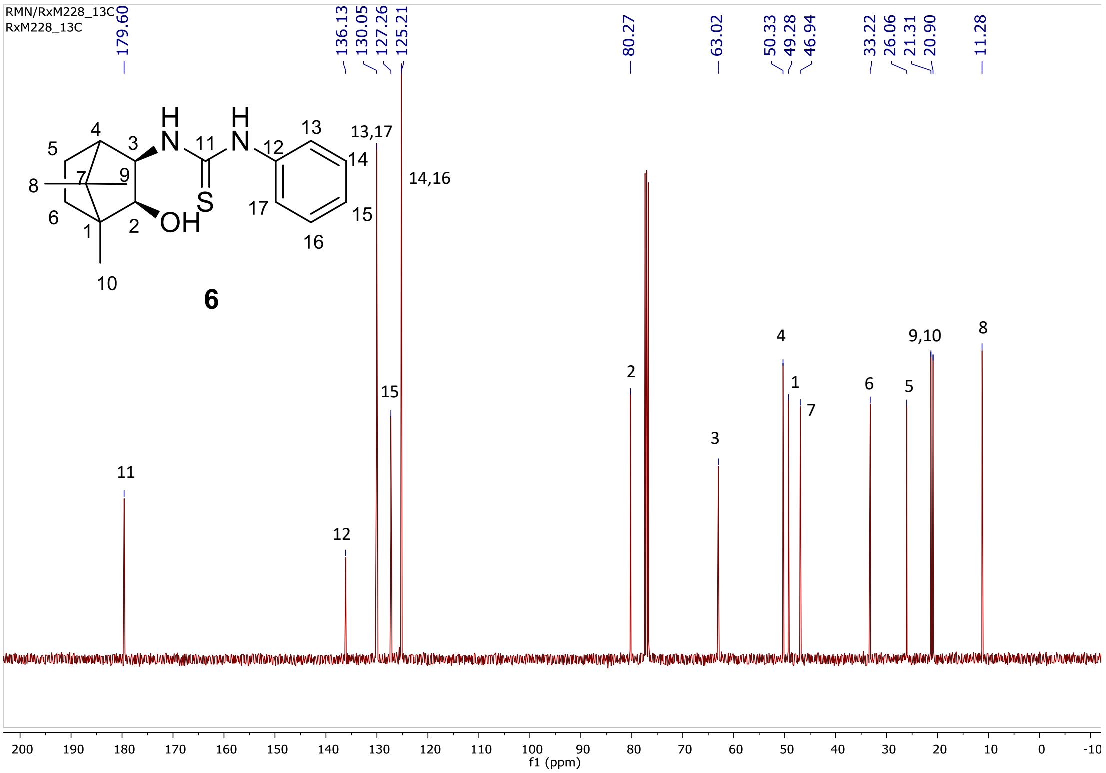

Figure S18. HRMS of 1-((1*R*,2*S*,3*R*,4*S*)-2-Hydroxy-1,7,7-trimethylbicyclo[2.2.1]heptan-3-yl)-3-phenylthiourea **6**

```
[ Elemental Composition ]
Data  : 031-LEM-HR                    Date : 23-Jun-2023 13:54

Sample: RxM228              Operator name M.en ITA Victoria Labastida G.
Note : UDLAP Mildred López  Centro de Investigaciones Químicas UAEM

Inlet : Direct                        Ion Mode : FAB+
RT : 0.08 min                         Scan#: 3+(45,49)

Elements : C 40/0, H 49/0, O 1/0, N 2/0, S 1/0
Mass Tolerance      : 1000ppm, 3mmu if m/z < 3, 5mmu if m/z > 5

Unsaturation (U.S.) : -0.5 - 10.0

Observed m/z Int%   Err[ppm / mmu]    U.S.  Composition
  305.1726   100.0    +12.6 /   +3.8    7.5  C 17 H 25 O N 2 S
```

X-ray diffraction data for thioureas 1 and 6

1-((1*R*,2*S*,3*R*,4*S*)-2-Hydroxy-1,7,7-trimethylbicyclo[2.2.1]heptan-3-yl)-3-((*S*)-1-phenylethyl)thiourea-**1**

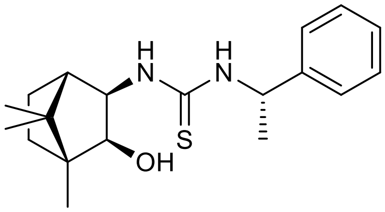

(1*R*,2*S*,3*R*,4*S*,12*S*)-**1**

Table S1. Crystal data and structure refinement for 1-((1*R*,2*S*,3*R*,4*S*)-2-Hydroxy-1,7,7-trimethylbicyclo[2.2.1]heptan-3-yl)-3-((*S*)-1-phenylethyl)thiourea-**1**

Identification code	RxM83-C3_B_Mo
Empirical formula	$C_{19}H_{28}N_2OS$
Formula weight	332.49
Temperature/K	293(2)
Crystal system	monoclinic
Space group	$P2_1$
a/Å	7.6886(3)
b/Å	27.9174(10)
c/Å	8.9652(4)
α/°	90
β/°	101.610(4)
γ/°	90
Volume/Å^3	1884.97(13)
Z	4
ρ_{calc}g/cm^3	1.172
μ/mm^{-1}	0.178
F(000)	720.0
Crystal size/mm^3	0.526 × 0.246 × 0.195
Radiation	Mo Kα (λ = 0.71073)
2Θ range for data collection/°	5.838 to 61.012
Index ranges	-10 ≤ h ≤ 10, -39 ≤ k ≤ 39, -12 ≤ l ≤ 12
Reflections collected	25311
Independent reflections	11162 [R_{int} = 0.0581, R_{sigma} = 0.0727]
Data/restraints/parameters	11162/1/425
Goodness-of-fit on F^2	1.005
Final R indexes [I>=2σ (I)]	R_1 = 0.0549, wR_2 = 0.1220
Final R indexes [all data]	R_1 = 0.0821, wR_2 = 0.1392
Largest diff. peak/hole / e Å^{-3}	0.40/-0.19

Flack parameter -0.04(4)

Table S2. Fractional Atomic Coordinates (×104) and Equivalent Isotropic Displacement Parameters (Å2×103) for **1**. Ueq is defined as 1/3 of the trace of the orthogonalised UIJ tensor.

Atom	x	y	z	U(eq)
S1	4952.4(12)	4947.2(3)	6392.6(10)	40.8(2)
S2	10111.2(13)	5282.9(4)	2901.6(11)	51.4(3)
O2	5175(3)	6055.9(9)	5237(3)	45.2(6)
N2	6305(3)	4258.6(9)	4907(3)	34.7(6)
N5	8175(3)	5803.5(10)	4443(3)	37.0(6)
N6	10629(4)	5447.7(10)	5872(3)	39.3(6)
O1	6011(4)	3909.7(12)	7805(4)	68.5(9)
C20	4334(4)	6449.4(11)	2726(4)	36.5(7)
C33	10408(4)	6136.3(12)	7599(4)	36.6(7)
N3	3722(4)	4600.0(11)	3635(4)	48.6(8)
C31	10213(5)	5600.2(12)	7324(4)	40.3(7)
C3	7937(4)	4229.2(11)	6078(4)	33.0(6)
C25	3845(5)	6278.3(14)	1062(4)	47.3(8)
C22	6770(4)	5877.4(11)	3133(4)	34.3(6)
C21	4970(4)	5991.3(11)	3630(4)	35.2(7)
C34	11634(5)	6405.8(13)	7027(4)	45.6(8)
C23	6973(4)	6299.2(13)	2078(4)	39.6(7)
C30	9604(4)	5526.5(10)	4502(4)	33.5(6)
C7	8820(5)	3388.0(12)	6344(5)	45.1(8)
C38	9381(5)	6362.2(16)	8499(5)	52.6(9)
C11	5016(4)	4577.5(11)	4865(4)	36.4(7)
C26	6086(5)	6729.1(12)	2740(4)	39.6(7)
C24	5641(5)	6191.1(16)	593(4)	51.4(9)
C32	11385(6)	5318.1(14)	8622(4)	57.2(10)
C15	4890(6)	3594.2(14)	1385(5)	51.1(9)
C19	2699(6)	3579.4(17)	2894(5)	59.8(11)
C4	9248(5)	3860.9(13)	5602(4)	44.8(8)
C14	3788(5)	3841.9(13)	2148(4)	40.6(7)
C1	9020(6)	3615.7(15)	7961(5)	53.2(9)
C16	4887(7)	3090.8(15)	1373(6)	66.1(13)
C12	3678(5)	4391.7(13)	2117(4)	46.2(8)
C2	7754(5)	4046.4(14)	7675(4)	48.3(9)
C18	2738(7)	3077.4(18)	2886(6)	72.8(14)
C28	5839(7)	7169.6(15)	1671(6)	65.3(12)
C29	2843(5)	6710.0(15)	3263(5)	56.1(10)

C27	7066(6)	6905.5(13)	4300(5)	53.0(10)
C37	9574(6)	6848.2(17)	8818(6)	66.0(12)
C8	6954(6)	3182.0(14)	5694(6)	68.2(13)
C17	3830(7)	2839.9(16)	2131(6)	70.5(13)
C5	11059(5)	3983.8(18)	6544(5)	61.4(11)
C35	11806(7)	6892.1(15)	7330(5)	61.0(11)
C36	10758(7)	7111.9(15)	8218(6)	67.6(13)
C13	4970(7)	4641.2(15)	1309(5)	68.2(13)
C6	10940(5)	3832.8(17)	8165(5)	59.8(11)
C9	10133(7)	2981.0(18)	6241(7)	76.6(14)
C10	8741(8)	3289(2)	9240(7)	88.9(18)

Table S3. Anisotropic Displacement Parameters (Å^2×10^3) for **1**. The Anisotropic displacement factor exponent takes the form: $-2\pi^2[h^2a^{*2}U_{11}+2hka^*b^*U_{12}+\ldots]$.

Atom	U_{11}	U_{22}	U_{33}	U_{23}	U_{13}	U_{12}
S1	48.5(5)	35.9(4)	36.5(4)	-5.7(4)	5.2(4)	10.1(4)
S2	44.4(5)	69.7(6)	37.3(5)	-12.5(5)	1.9(4)	23.5(4)
O2	52.7(15)	44.0(12)	43.4(14)	8.7(11)	20.5(12)	10.7(11)
N2	38.0(14)	34.3(12)	29.1(13)	-4.6(11)	0.4(11)	10.0(11)
N5	34.9(13)	42.4(14)	31.6(14)	-5.1(12)	1.3(11)	11.6(11)
N6	38.4(15)	41.8(14)	34.5(15)	-3.9(12)	0.0(12)	11.8(12)
O1	61.3(18)	83(2)	71(2)	29.4(18)	35.5(17)	18.1(16)
C20	29.7(15)	38.6(15)	40.8(18)	3.9(14)	5.9(13)	6.5(13)
C33	36.5(16)	40.5(16)	31.1(16)	-1.9(14)	2.8(13)	9.0(13)
N3	42.7(16)	56.1(17)	41.0(17)	-14.1(15)	-6.0(13)	24.0(14)
C31	42.5(18)	42.9(17)	34.8(18)	-2.4(15)	6.0(14)	1.6(15)
C3	32.3(15)	30.0(13)	35.6(16)	2.4(13)	4.0(12)	1.5(12)
C25	41.2(19)	56(2)	41(2)	0.1(17)	-0.3(16)	6.9(16)
C22	29.6(15)	34.3(14)	37.2(16)	-3.9(13)	2.5(12)	4.9(12)
C21	30.3(15)	31.0(14)	44.9(18)	1.2(14)	8.7(13)	1.0(12)
C34	56(2)	43.0(17)	41(2)	-3.5(16)	17.2(17)	-0.7(16)
C23	29.5(15)	54.4(19)	35.1(17)	3.2(15)	7.0(13)	3.9(14)
C30	35.4(16)	30.9(14)	31.8(16)	-4.0(13)	1.5(12)	5.9(12)
C7	41.8(19)	35.5(16)	58(2)	5.6(16)	9.5(17)	7.7(14)
C38	45(2)	62(2)	52(2)	-7.9(19)	15.0(18)	11.0(18)
C11	38.1(17)	34.5(15)	35.9(17)	-1.0(14)	5.7(14)	7.1(13)
C26	40.5(18)	35.4(15)	41.6(18)	8.1(14)	5.1(14)	-1.6(13)
C24	52(2)	67(2)	32.8(18)	-4.8(17)	2.9(16)	10.2(18)
C32	83(3)	44.2(19)	37.8(19)	0.6(17)	-3.2(19)	12(2)
C15	61(2)	46.8(19)	49(2)	-3.1(18)	20.0(19)	-2.2(18)

Table S3. Anisotropic Displacement Parameters (Å^2×10^3) for **1**. The Anisotropic displacement factor exponent takes the form: $-2\pi^2[h^2a^{*2}U_{11}+2hka^*b^*U_{12}+\ldots]$.

Atom	U_{11}	U_{22}	U_{33}	U_{23}	U_{13}	U_{12}
C19	48(2)	78(3)	57(3)	-14(2)	19.5(19)	-7(2)
C4	40.7(18)	54(2)	41.2(19)	0.2(17)	12.0(15)	7.8(16)
C14	39.9(18)	46.1(17)	34.1(17)	-8.0(15)	3.2(14)	1.3(14)
C1	56(2)	55(2)	47(2)	14.8(19)	5.1(18)	9.3(18)
C16	88(3)	46(2)	67(3)	-17(2)	24(3)	7(2)
C12	48(2)	50.7(19)	33.3(17)	-6.3(16)	-7.3(15)	15.0(16)
C2	51(2)	56(2)	38.1(19)	9.0(17)	9.0(16)	8.4(17)
C18	74(3)	75(3)	71(3)	-2(3)	18(3)	-35(3)
C28	82(3)	49(2)	65(3)	23(2)	15(2)	-1(2)
C29	50(2)	58(2)	65(3)	11(2)	21.0(19)	22.9(18)
C27	64(2)	36.2(17)	53(2)	-5.2(17)	-0.4(19)	-8.2(17)
C37	65(3)	65(3)	67(3)	-14(2)	11(2)	27(2)
C8	65(3)	42.1(19)	92(4)	-6(2)	0(2)	-0.8(19)
C17	88(3)	48(2)	71(3)	-11(2)	5(3)	-15(2)
C5	36.3(19)	76(3)	69(3)	3(2)	3.2(19)	8.7(19)
C35	84(3)	47(2)	51(3)	2.4(19)	11(2)	-6(2)
C36	92(4)	43(2)	58(3)	-10(2)	-8(3)	16(2)
C13	112(4)	48(2)	43(2)	2.1(19)	10(2)	0(2)
C6	48(2)	68(3)	55(2)	2(2)	-11.3(18)	8(2)
C9	69(3)	54(2)	103(4)	-2(3)	9(3)	24(2)
C10	99(4)	92(4)	81(4)	44(3)	31(3)	31(3)

Table S4. Bond Lengths for **1.**

Atom	Atom	Length/Å	Atom	Atom	Length/Å
S1	C11	1.724(3)	C34	C35	1.386(5)
S2	C30	1.702(3)	C23	C26	1.556(5)
O2	C21	1.429(4)	C23	C24	1.538(5)
N2	C3	1.467(4)	C7	C4	1.544(5)
N2	C11	1.327(4)	C7	C1	1.561(6)
N5	C22	1.441(4)	C7	C8	1.547(6)
N5	C30	1.336(4)	C7	C9	1.535(5)
N6	C31	1.465(4)	C38	C37	1.388(6)
N6	C30	1.337(4)	C26	C28	1.547(5)
O1	C2	1.421(5)	C26	C27	1.531(5)
C20	C25	1.539(5)	C15	C14	1.377(5)
C20	C21	1.540(4)	C15	C16	1.405(6)
C20	C26	1.554(5)	C19	C14	1.382(5)

Table S4. Bond Lengths for **1.**

Atom	Atom	Length/Å	Atom	Atom	Length/Å
C20	C29	1.516(5)	C19	C18	1.402(7)
C33	C31	1.519(5)	C4	C5	1.515(6)
C33	C34	1.383(5)	C14	C12	1.537(5)
C33	C38	1.389(5)	C1	C2	1.536(5)
N3	C11	1.330(4)	C1	C6	1.572(6)
N3	C12	1.474(5)	C1	C10	1.515(6)
C31	C32	1.538(5)	C16	C17	1.354(7)
C3	C4	1.558(4)	C12	C13	1.512(6)
C3	C2	1.553(5)	C18	C17	1.353(7)
C25	C24	1.542(5)	C37	C36	1.363(7)
C22	C21	1.570(4)	C5	C6	1.534(6)
C22	C23	1.538(5)	C35	C36	1.385(7)

Table S5. Bond Angles for **1.**

Atom	Atom	Atom	Angle/°	Atom	Atom	Atom	Angle/°
C11	N2	C3	125.8(3)	C37	C38	C33	120.9(4)
C30	N5	C22	126.2(3)	N2	C11	S1	121.3(3)
C30	N6	C31	125.1(3)	N2	C11	N3	119.0(3)
C25	C20	C21	104.0(3)	N3	C11	S1	119.6(2)
C25	C20	C26	101.9(3)	C20	C26	C23	93.2(2)
C21	C20	C26	103.3(2)	C28	C26	C20	113.3(3)
C29	C20	C25	113.8(3)	C28	C26	C23	112.7(3)
C29	C20	C21	114.3(3)	C27	C26	C20	115.8(3)
C29	C20	C26	117.8(3)	C27	C26	C23	115.1(3)
C34	C33	C31	121.9(3)	C27	C26	C28	106.7(3)
C34	C33	C38	118.6(3)	C23	C24	C25	102.1(3)
C38	C33	C31	119.5(3)	C14	C15	C16	120.4(4)
C11	N3	C12	128.4(3)	C14	C19	C18	120.8(4)
N6	C31	C33	113.5(3)	C7	C4	C3	103.9(3)
N6	C31	C32	108.9(3)	C5	C4	C3	105.7(3)
C33	C31	C32	111.0(3)	C5	C4	C7	101.8(3)
N2	C3	C4	110.4(3)	C15	C14	C19	117.9(4)
N2	C3	C2	117.0(3)	C15	C14	C12	122.0(3)
C2	C3	C4	102.7(3)	C19	C14	C12	120.1(3)
C20	C25	C24	104.8(3)	C7	C1	C6	100.4(3)
N5	C22	C21	110.9(3)	C2	C1	C7	102.9(3)
N5	C22	C23	117.3(3)	C2	C1	C6	105.4(3)
C23	C22	C21	102.9(2)	C10	C1	C7	117.1(4)

Table S5. Bond Angles for **1.**

Atom	Atom	Atom	Angle/°	Atom	Atom	Atom	Angle/°
O2	C21	C20	112.6(3)	C10	C1	C2	114.6(4)
O2	C21	C22	112.8(3)	C10	C1	C6	114.6(4)
C20	C21	C22	102.9(2)	C17	C16	C15	120.9(4)
C33	C34	C35	120.4(4)	N3	C12	C14	112.7(3)
C22	C23	C26	104.5(2)	N3	C12	C13	111.4(3)
C24	C23	C22	104.5(3)	C13	C12	C14	115.4(3)
C24	C23	C26	102.4(3)	O1	C2	C3	115.2(3)
N5	C30	S2	121.6(2)	O1	C2	C1	110.7(3)
N5	C30	N6	117.4(3)	C1	C2	C3	103.8(3)
N6	C30	S2	121.0(2)	C17	C18	C19	120.6(4)
C4	C7	C1	93.6(3)	C36	C37	C38	119.9(4)
C4	C7	C8	114.7(3)	C18	C17	C16	119.5(4)
C8	C7	C1	114.3(3)	C4	C5	C6	104.1(3)
C9	C7	C4	114.0(3)	C36	C35	C34	120.2(4)
C9	C7	C1	114.5(4)	C37	C36	C35	120.0(4)
C9	C7	C8	105.9(3)	C5	C6	C1	103.2(3)

Table S6. Torsion Angles for **1**.

A	B	C	D	Angle/°	A	B	C	D	Angle/°
N2	C3	C4	C7	-92.9(3)	C11	N3	C12	C14	62.7(5)
N2	C3	C4	C5	160.3(3)	C11	N3	C12	C13	-69.0(5)
N2	C3	C2	O1	2.3(4)	C26	C20	C25	C24	33.0(3)
N2	C3	C2	C1	123.4(3)	C26	C20	C21	O2	84.9(3)
N5	C22	C21	O2	7.5(4)	C26	C20	C21	C22	-36.8(3)
N5	C22	C21	C20	129.1(3)	C26	C23	C24	C25	-37.6(4)
N5	C22	C23	C26	-90.0(3)	C24	C23	C26	C20	56.2(3)
N5	C22	C23	C24	162.8(3)	C24	C23	C26	C28	-60.7(4)
C20	C25	C24	C23	2.7(4)	C24	C23	C26	C27	176.7(3)
C33	C34	C35	C36	0.4(7)	C15	C14	C12	N3	-134.6(4)
C33	C38	C37	C36	1.4(7)	C15	C14	C12	C13	-5.0(5)
C31	N6	C30	S2	-173.5(3)	C15	C16	C17	C18	1.2(8)
C31	N6	C30	N5	6.6(5)	C19	C14	C12	N3	48.6(5)
C31	C33	C34	C35	-178.5(4)	C19	C14	C12	C13	178.2(4)
C31	C33	C38	C37	177.6(4)	C19	C18	C17	C16	-0.2(8)
C3	N2	C11	S1	-10.7(4)	C4	C3	C2	O1	-118.8(3)
C3	N2	C11	N3	171.5(3)	C4	C3	C2	C1	2.4(4)
C3	C4	C5	C6	70.7(4)	C4	C7	C1	C2	54.0(3)
C25	C20	C21	O2	-169.0(3)	C4	C7	C1	C6	-54.7(3)

Table S6. Torsion Angles for **1**.

A	B	C	D	Angle/°	A	B	C	D	Angle/°
C25	C20	C21	C22	69.3(3)	C4	C7	C1	C10	-179.4(4)
C25	C20	C26	C23	-53.6(3)	C4	C5	C6	C1	1.9(4)
C25	C20	C26	C28	62.8(4)	C14	C15	C16	C17	-1.2(8)
C25	C20	C26	C27	-173.5(3)	C14	C19	C18	C17	-0.8(8)
C22	N5	C30	S2	9.6(5)	C1	C7	C4	C3	-52.5(3)
C22	N5	C30	N6	-170.5(3)	C1	C7	C4	C5	57.1(3)
C22	C23	C26	C20	-52.5(3)	C16	C15	C14	C19	0.2(6)
C22	C23	C26	C28	-169.5(3)	C16	C15	C14	C12	-176.7(4)
C22	C23	C26	C27	68.0(4)	C12	N3	C11	S1	167.0(3)
C22	C23	C24	C25	71.1(3)	C12	N3	C11	N2	-15.1(5)
C21	C20	C25	C24	-74.2(3)	C2	C3	C4	C7	32.6(4)
C21	C20	C26	C23	54.1(3)	C2	C3	C4	C5	-74.2(4)
C21	C20	C26	C28	170.5(3)	C2	C1	C6	C5	-72.8(4)
C21	C20	C26	C27	-65.8(4)	C18	C19	C14	C15	0.8(6)
C21	C22	C23	C26	32.1(3)	C18	C19	C14	C12	177.7(4)
C21	C22	C23	C24	-75.1(3)	C29	C20	C25	C24	160.8(3)
C34	C33	C31	N6	-30.8(5)	C29	C20	C21	O2	-44.3(4)
C34	C33	C31	C32	92.2(4)	C29	C20	C21	C22	-166.0(3)
C34	C33	C38	C37	0.1(6)	C29	C20	C26	C23	-178.9(3)
C34	C35	C36	C37	1.1(7)	C29	C20	C26	C28	-62.5(4)
C23	C22	C21	O2	-118.8(3)	C29	C20	C26	C27	61.3(4)
C23	C22	C21	C20	2.9(3)	C8	C7	C4	C3	66.4(4)
C30	N5	C22	C21	151.2(3)	C8	C7	C4	C5	176.0(4)
C30	N5	C22	C23	-91.0(4)	C8	C7	C1	C2	-65.2(4)
C30	N6	C31	C33	-72.9(4)	C8	C7	C1	C6	-173.9(3)
C30	N6	C31	C32	163.0(3)	C8	C7	C1	C10	61.4(5)
C7	C4	C5	C6	-37.6(4)	C6	C1	C2	O1	-167.1(3)
C7	C1	C2	O1	88.1(4)	C6	C1	C2	C3	68.7(4)
C7	C1	C2	C3	-36.1(4)	C9	C7	C4	C3	-171.4(4)
C7	C1	C6	C5	33.8(4)	C9	C7	C4	C5	-61.7(4)
C38	C33	C31	N6	151.8(3)	C9	C7	C1	C2	172.4(3)
C38	C33	C31	C32	-85.2(4)	C9	C7	C1	C6	63.8(4)
C38	C33	C34	C35	-1.0(6)	C9	C7	C1	C10	-60.9(5)
C38	C37	C36	C35	-2.0(7)	C10	C1	C2	O1	-40.1(5)
C11	N2	C3	C4	-171.2(3)	C10	C1	C2	C3	-164.3(4)
C11	N2	C3	C2	71.9(4)	C10	C1	C6	C5	160.2(4)

Table S7. Hydrogen Atom Coordinates (Å×10^4) and Isotropic Displacement Parameters (Å^2×10^3) for **1**.

**Atom**	***x***	***y***	***z***	**U(eq)**
H2B	4972.27	5802.22	5630.89	68
H2	6171.38	4050.8	4184.84	42
H5	8085.07	5951.98	5264.19	44
H6	11609.07	5295.77	5902.79	47
H1	5345.73	4141.38	7625.47	103
H3	2789.96	4756.45	3738.49	58
H31	8973.12	5515.08	7311.16	48
H3A	8504.83	4545.03	6189.56	40
H25A	3163.7	6520.75	421.21	57
H25B	3153.69	5985.39	984.09	57
H22	6621.58	5582.76	2526.69	41
H21	4123.06	5731.61	3296.6	42
H34	12347.36	6259.85	6435.65	55
H23	8191.62	6358.13	1951.25	47
H38	8551.92	6185.48	8892.72	63
H24A	5746.22	5862.77	268.5	62
H24B	5803.47	6406.17	-218.07	62
H32A	11243.89	4981.05	8421.42	86
H32B	11037.42	5391.47	9566.54	86
H32C	12605.68	5405.62	8688.26	86
H15	5640.47	3761.01	874.81	61
H19	1931.59	3737.77	3407.74	72
H4	9201.22	3841.11	4503.01	54
H16	5621.17	2927.72	838.29	79
H12	2496.21	4467.04	1520.43	55
H2A	8183.73	4294.92	8431.57	58
H18	2007.79	2906.11	3404.39	87
H28A	5143.36	7080.48	697.89	98
H28B	6979.59	7282.79	1545.24	98
H28C	5240.61	7418.95	2107.55	98
H29A	2519.17	6989.96	2648.15	84
H29B	3231.53	6803.32	4307.9	84
H29C	1833.6	6501.77	3173.98	84
H27A	6456.79	7177.94	4601.27	79
H27B	8256.17	6995.03	4242.24	79
H27C	7100.09	6654.14	5036.51	79
H37	8896.19	6993.56	9441.13	79
H8A	6070.22	3418.25	5772.49	102
H8B	6761.62	2901.99	6260.55	102

Table S7. Hydrogen Atom Coordinates (Å×10^4) and Isotropic Displacement Parameters (Å^2×10^3) for **1**.

**Atom**	***x***	***y***	***z***	**U(eq)**
H8C	6873.15	3097.74	4643.54	102
H17	3853.96	2506.88	2131.52	85
H5A	11295.79	4324.13	6497.62	74
H5B	11986.78	3807.89	6191.78	74
H35	12627.91	7071.51	6936.18	73
H36	10861.98	7439.35	8404.9	81
H13A	4711.16	4977.77	1240.89	102
H13B	4860.71	4510.82	303.57	102
H13C	6159.54	4593.26	1869.86	102
H6A	11080.71	4106.19	8846.78	72
H6B	11835.71	3595.38	8560.1	72
H9A	9927.58	2860.4	5217.56	115
H9B	9967.98	2727.79	6923.35	115
H9C	11326.1	3100.28	6516.98	115
H10A	7526.86	3184.64	9055.97	133
H10B	9013.53	3459.13	10188.55	133
H10C	9506.9	3015.14	9286.61	133

1-((1*R*,2*S*,3*R*,4*S*)-2-Hydroxy-1,7,7-trimethylbicyclo[2.2.1]heptan-3-yl)-3-phenylthiourea **6**

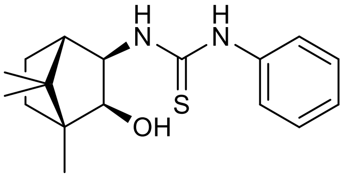

(1*R*,2*S*,3*R*,4*S*)-**6**

Table S8. Crystal data and structure refinement for **6**

Identification code	Cat-5_Mo
Empirical formula	$C_{17}H_{24}N_2OS$
Formula weight	304.44
Temperature/K	293(2)
Crystal system	monoclinic
Space group	$P2_1$
a/Å	7.6667(2)
b/Å	11.9881(2)
c/Å	18.6749(4)
α/°	90

β/°	100.548(2)
γ/°	90
Volume/Å^3	1687.39(6)
Z	4
ρ_{calc}g/cm^3	1.198
μ/mm^{-1}	0.193
F(000)	656.0
Crystal size/mm^3	0.52 × 0.33 × 0.21
Radiation	Mo Kα (λ = 0.71073)
2Θ range for data collection/°	6.208 to 61.012
Index ranges	-10 ≤ h ≤ 10, -17 ≤ k ≤ 17, -26 ≤ l ≤ 26
Reflections collected	54301
Independent reflections	10257 [R_{int} = 0.0384, R_{sigma} = 0.0291]
Data/restraints/parameters	10257/97/387
Goodness-of-fit on F^2	1.016
Final R indexes [I>=2σ (I)]	R_1 = 0.0436, wR_2 = 0.1110
Final R indexes [all data]	R_1 = 0.0665, wR_2 = 0.1248
Largest diff. peak/hole / e Å^{-3}	0.19/-0.14
Flack parameter	-0.04(2)

Table S9. Fractional Atomic Coordinates (×10^4) and Equivalent Isotropic Displacement Parameters (Å^2×10^3) for **6.** U_{eq} is defined as 1/3 of the trace of the orthogonalised U_{IJ} tensor.

**Atom**	***x***	***y***	***z***	**U(eq)**
S1	2370.2(9)	5626.3(5)	3208.3(4)	52.91(17)
S2	7457.9(9)	4460.9(6)	1741.4(5)	60.2(2)
O2	2246(3)	7177.6(17)	1733.0(11)	54.9(5)
O1	3945(3)	4231(2)	4683.0(13)	75.6(7)
N4	5339(3)	6224.7(18)	1693.2(13)	48.9(5)
N3	8070(3)	6397(2)	2420.1(15)	58.6(6)
N1	4250(3)	3756(2)	3253.9(13)	52.3(5)
N2	1559(3)	3713(2)	2499.8(14)	58.0(6)
C18	6909(3)	5769(2)	1957.1(14)	45.6(5)
C2	5618(4)	4491(3)	4505.3(16)	56.4(6)
C25	2808(4)	6788(2)	145.9(15)	48.6(6)
C12	1795(4)	2572(2)	2309.5(16)	53.4(6)
C29	7726(3)	7507(3)	2630.6(16)	52.1(6)
C19	3895(3)	5662(2)	1224.5(13)	42.7(5)
C24	2061(3)	6189(2)	1291.9(14)	44.4(5)
C23	1212(3)	6424(2)	495.6(14)	44.0(5)
C11	2749(3)	4285(2)	2970.9(14)	48.5(6)

Table S9. Fractional Atomic Coordinates (×10[4]) and Equivalent Isotropic Displacement Parameters (Å[2]×10[3]) for **6.** U_{eq} is defined as 1/3 of the trace of the orthogonalised U_{IJ} tensor.

Atom	*x*	*y*	*z*	U(eq)
C22	792(4)	5250(2)	162.9(17)	54.1(6)
C20	3891(3)	5726(2)	400.5(15)	47.4(5)
C21	2623(4)	4788(2)	73.3(17)	55.8(7)
C27	3697(5)	7883(3)	423(2)	66.6(8)
C1	7139(4)	3812(3)	4961.5(17)	62.5(7)
C28	2301(6)	6894(3)	-692.8(18)	75.6(9)
C7	7182(4)	2758(2)	4502.8(17)	56.3(6)
C3	5803(3)	4234(2)	3706.4(15)	51.0(6)
C34	7539(5)	8366(3)	2125(2)	70.9(8)
C26	-399(4)	7183(3)	413.2(19)	66.9(8)
C15	2294(5)	372(3)	1949(3)	83.2(11)
C4	7366(4)	3402(3)	3812.1(18)	62.2(8)
C13	1072(5)	1738(3)	2654(3)	81.4(10)
C30	7554(5)	7736(4)	3333(2)	76.7(10)
C14	1340(6)	621(4)	2482(3)	95.3(12)
C31	7179(5)	8854(5)	3516(3)	93.0(14)
C17	2766(6)	2306(3)	1788(2)	76.1(10)
C32	6979(6)	9664(4)	2995(3)	93.0(13)
C33	7144(6)	9424(3)	2316(3)	89.3(11)
C8	5519(6)	2026(3)	4435(3)	89.7(12)
C9	8774(6)	1993(4)	4771(3)	90.1(12)
C16	3023(6)	1213(4)	1615(2)	86.2(11)
C6	8834(5)	4397(4)	4848(3)	105.9(17)
C10	6988(8)	3662(6)	5757(2)	128(2)
C5	9016(4)	4081(5)	4055(3)	99.1(15)

Table S10. Anisotropic Displacement Parameters (Å[2]×10[3]) for **6**. The Anisotropic displacement factor exponent takes the form: $-2\pi^2[h^2a^{*2}U_{11}+2hka^*b^*U_{12}+\ldots]$.

Atom	U_{11}	U_{22}	U_{33}	U_{23}	U_{13}	U_{12}
S1	55.1(4)	45.2(3)	55.2(4)	3.4(3)	1.6(3)	8.7(3)
S2	48.8(3)	48.2(4)	77.0(5)	-9.8(3)	-5.7(3)	12.3(3)
O2	59.8(11)	53.0(11)	51.8(11)	-4.9(8)	10.2(9)	8.6(9)
O1	66.7(12)	101.6(19)	61.1(13)	9.5(12)	18.7(10)	24.4(13)
N4	41.2(10)	38.2(11)	63.2(13)	-6.4(10)	-1.1(9)	3.9(8)
N3	42.8(11)	54.8(14)	70.4(16)	-12.0(12)	-9.8(10)	6.1(10)
N1	47.3(11)	50.0(13)	55.1(12)	-5.1(10)	-2.8(9)	9.9(10)

Table S10. Anisotropic Displacement Parameters ($Å^2×10^3$) for **6**. The Anisotropic displacement factor exponent takes the form: $-2\pi^2[h^2a^{*2}U_{11}+2hka^*b^*U_{12}+…]$.

Atom	U_{11}	U_{22}	U_{33}	U_{23}	U_{13}	U_{12}
N2	47.9(11)	54.1(14)	63.7(14)	-7.4(11)	-11.4(10)	13.4(10)
C18	40.9(11)	43.3(13)	50.7(13)	0.9(11)	3.2(9)	1.3(10)
C2	56.0(14)	46.5(14)	61.2(16)	-7.2(12)	-3.9(12)	7.5(12)
C25	52.3(14)	44.4(13)	49.5(14)	6.1(11)	10.4(11)	-2.5(11)
C12	45.9(13)	51.0(15)	56.9(16)	-2.9(12)	-7.9(11)	5.5(11)
C29	37.1(12)	55.9(16)	60.1(16)	-13.5(12)	0.3(10)	-2.6(11)
C19	36.5(10)	34.2(10)	54.6(13)	0.3(10)	1.1(9)	1.4(9)
C24	39.9(11)	41.0(12)	52.5(14)	4.4(10)	8.9(10)	1.1(9)
C23	38.0(11)	41.0(12)	51.9(14)	4.8(10)	5.1(10)	5.2(9)
C11	45.1(12)	52.3(15)	45.9(13)	4.4(11)	2.5(10)	6.7(11)
C22	45.0(13)	52.5(15)	60.6(16)	-1.2(12)	-1.4(11)	-5.0(11)
C20	38.9(11)	46.9(13)	57.4(14)	-6.2(12)	11.2(10)	-0.4(11)
C21	56.5(15)	45.8(14)	60.9(17)	-9.1(12)	-0.6(13)	1.3(11)
C27	73.8(19)	46.2(15)	79(2)	10.3(14)	12.8(16)	-11.9(14)
C1	58.2(16)	68.7(19)	54.7(16)	-5.4(14)	-5.7(12)	10.1(14)
C28	96(3)	77(2)	55.0(18)	14.4(16)	15.4(17)	-3.8(19)
C7	50.4(14)	48.9(14)	66.8(17)	6.6(13)	3.3(12)	9.6(11)
C3	41.5(12)	52.7(15)	57.2(15)	13.3(12)	5.2(10)	0.4(11)
C34	84(2)	58.4(18)	71(2)	-8.4(15)	17.1(17)	-6.4(17)
C26	54.5(16)	72(2)	70.0(19)	3.7(16)	0.4(13)	23.1(15)
C15	78(2)	55.4(19)	107(3)	-10.3(18)	-7(2)	7.1(16)
C4	39.8(12)	85(2)	61.8(17)	5.4(15)	10.2(11)	12.6(14)
C13	73(2)	70(2)	106(3)	-4.8(19)	29(2)	-4.5(18)
C30	74(2)	95(3)	57.3(18)	-16.4(18)	2.5(16)	-9.8(19)
C14	97(3)	61(2)	125(4)	8(2)	13(2)	-13(2)
C31	72(2)	128(4)	77(2)	-54(3)	8.9(19)	-5(2)
C17	104(3)	59(2)	68(2)	2.2(15)	23.2(19)	4.1(18)
C32	82(3)	77(3)	115(3)	-37(3)	4(2)	4(2)
C33	106(3)	56.0(19)	101(3)	-6(2)	9(2)	-2(2)
C8	87(3)	48.2(18)	129(4)	17(2)	6(2)	-5.6(18)
C9	80(2)	75(2)	107(3)	13(2)	-5(2)	32(2)
C16	105(3)	74(2)	81(3)	-15(2)	21(2)	13(2)
C6	63(2)	75(3)	157(5)	-6(3)	-40(2)	-13.6(19)
C10	136(4)	192(6)	49(2)	-7(3)	-4(2)	76(4)
C5	43.8(16)	128(4)	122(4)	48(3)	7.4(18)	-10.5(19)

Table S11. Bond Lengths for **6.**

Atom	Atom	Length/Å	Atom	Atom	Length/Å
S1	C11	1.707(3)	C19	C20	1.540(4)
S2	C18	1.691(3)	C24	C23	1.536(4)
O2	C24	1.435(3)	C23	C22	1.547(4)
O1	C2	1.418(4)	C23	C26	1.519(4)
N4	C18	1.331(3)	C22	C21	1.547(4)
N4	C19	1.446(3)	C20	C21	1.538(4)
N3	C18	1.351(3)	C1	C7	1.530(4)
N3	C29	1.425(4)	C1	C6	1.525(6)
N1	C11	1.335(3)	C1	C10	1.522(5)
N1	C3	1.446(4)	C7	C4	1.532(4)
N2	C12	1.434(4)	C7	C8	1.534(5)
N2	C11	1.335(4)	C7	C9	1.536(4)
C2	C1	1.544(4)	C3	C4	1.544(4)
C2	C3	1.555(4)	C34	C33	1.367(5)
C25	C23	1.552(4)	C15	C14	1.371(7)
C25	C20	1.546(4)	C15	C16	1.357(6)
C25	C27	1.525(4)	C4	C5	1.503(5)
C25	C28	1.548(4)	C13	C14	1.401(6)
C12	C13	1.360(5)	C30	C31	1.426(7)
C12	C17	1.368(5)	C31	C32	1.364(7)
C29	C34	1.387(5)	C17	C16	1.373(6)
C29	C30	1.370(5)	C32	C33	1.329(6)
C19	C24	1.568(3)	C6	C5	1.559(7)

Table S12. Bond Angles for **6.**

Atom	Atom	Atom	Angle/°	Atom	Atom	Atom	Angle/°
C18	N4	C19	124.9(2)	N2	C11	N1	117.0(3)
C18	N3	C29	124.3(2)	C21	C22	C23	104.1(2)
C11	N1	C3	126.9(2)	C19	C20	C25	104.5(2)
C11	N2	C12	123.4(2)	C21	C20	C25	102.4(2)
N4	C18	S2	122.7(2)	C21	C20	C19	104.2(2)
N4	C18	N3	116.9(2)	C20	C21	C22	102.4(2)
N3	C18	S2	120.38(19)	C7	C1	C2	102.6(2)
O1	C2	C1	112.3(3)	C6	C1	C2	104.9(3)
O1	C2	C3	115.2(2)	C6	C1	C7	101.3(3)
C1	C2	C3	103.4(2)	C10	C1	C2	114.6(3)
C20	C25	C23	93.38(19)	C10	C1	C7	117.5(4)
C20	C25	C28	113.6(3)	C10	C1	C6	114.1(4)

Table S12. Bond Angles for **6.**

Atom	Atom	Atom	Angle/°	Atom	Atom	Atom	Angle/°
C27	C25	C23	115.9(3)	C1	C7	C4	94.0(2)
C27	C25	C20	115.0(2)	C1	C7	C8	114.5(3)
C27	C25	C28	106.7(2)	C1	C7	C9	114.1(3)
C28	C25	C23	112.2(3)	C4	C7	C8	115.0(3)
C13	C12	N2	120.2(3)	C4	C7	C9	112.7(3)
C13	C12	C17	119.2(3)	C8	C7	C9	106.5(3)
C17	C12	N2	120.6(3)	N1	C3	C2	115.4(2)
C34	C29	N3	120.4(3)	N1	C3	C4	110.8(3)
C30	C29	N3	120.4(3)	C4	C3	C2	102.1(2)
C30	C29	C34	119.2(3)	C33	C34	C29	120.7(3)
N4	C19	C24	111.2(2)	C16	C15	C14	119.4(4)
N4	C19	C20	116.3(2)	C7	C4	C3	104.2(2)
C20	C19	C24	102.84(19)	C5	C4	C7	102.7(3)
O2	C24	C19	112.5(2)	C5	C4	C3	106.2(3)
O2	C24	C23	112.6(2)	C12	C13	C14	120.3(4)
C23	C24	C19	102.92(19)	C29	C30	C31	118.5(4)
C24	C23	C25	103.39(19)	C15	C14	C13	119.6(4)
C24	C23	C22	104.0(2)	C32	C31	C30	120.0(4)
C22	C23	C25	102.1(2)	C12	C17	C16	120.6(4)
C26	C23	C25	118.5(2)	C33	C32	C31	120.6(4)
C26	C23	C24	113.6(2)	C32	C33	C34	121.0(4)
C26	C23	C22	113.5(2)	C15	C16	C17	120.8(4)
N1	C11	S1	121.3(2)	C1	C6	C5	104.3(3)
N2	C11	S1	121.7(2)	C4	C5	C6	101.6(3)

Table S13. Torsion Angles for **6.**

A	B	C	D	Angle/°	A	B	C	D	Angle/°
O2	C24	C23	C25	84.7(2)	C11	N1	C3	C4	-170.3(3)
O2	C24	C23	C22	-168.95(19)	C11	N2	C12	C13	96.1(4)
O2	C24	C23	C26	-45.1(3)	C11	N2	C12	C17	-83.1(4)
O1	C2	C1	C7	88.7(3)	C20	C25	C23	C24	54.0(2)
O1	C2	C1	C6	-165.8(3)	C20	C25	C23	C22	-53.8(2)
O1	C2	C1	C10	-39.8(5)	C20	C25	C23	C26	-179.2(3)
O1	C2	C3	N1	-0.5(4)	C20	C19	C24	O2	-118.5(2)
O1	C2	C3	C4	-120.8(3)	C20	C19	C24	C23	3.0(2)
N4	C19	C24	O2	6.7(3)	C27	C25	C23	C24	-66.0(3)
N4	C19	C24	C23	128.2(2)	C27	C25	C23	C22	-173.8(2)

Table S13. Torsion Angles for **6.**

A	B	C	D	Angle/°	A	B	C	D	Angle/°
N4	C19	C20	C25	-90.0(2)	C27	C25	C23	C26	60.8(4)
N4	C19	C20	C21	162.9(2)	C27	C25	C20	C19	68.5(3)
N3	C29	C34	C33	177.6(3)	C27	C25	C20	C21	177.0(3)
N3	C29	C30	C31	-178.9(3)	C1	C2	C3	N1	122.4(3)
N1	C3	C4	C7	-90.8(3)	C1	C2	C3	C4	2.1(3)
N1	C3	C4	C5	161.1(3)	C1	C7	C4	C3	-53.5(3)
N2	C12	C13	C14	-178.4(4)	C1	C7	C4	C5	57.1(3)
N2	C12	C17	C16	178.7(3)	C1	C6	C5	C4	2.4(4)
C18	N4	C19	C24	154.8(2)	C28	C25	C23	C24	171.2(2)
C18	N4	C19	C20	-87.9(3)	C28	C25	C23	C22	63.4(3)
C18	N3	C29	C34	-66.8(4)	C28	C25	C23	C26	-62.1(4)
C18	N3	C29	C30	112.4(3)	C28	C25	C20	C19	-168.2(2)
C2	C1	C7	C4	54.2(3)	C28	C25	C20	C21	-59.7(3)
C2	C1	C7	C8	-65.6(3)	C7	C1	C6	C5	33.2(4)
C2	C1	C7	C9	171.3(3)	C7	C4	C5	C6	-37.3(4)
C2	C1	C6	C5	-73.3(4)	C3	N1	C11	S1	-8.0(4)
C2	C3	C4	C7	32.7(3)	C3	N1	C11	N2	172.5(3)
C2	C3	C4	C5	-75.4(3)	C3	C2	C1	C7	-36.1(3)
C25	C23	C22	C21	32.9(3)	C3	C2	C1	C6	69.4(3)
C25	C20	C21	C22	-37.7(3)	C3	C2	C1	C10	-164.7(4)
C12	N2	C11	S1	-177.3(2)	C3	C4	C5	C6	71.8(4)
C12	N2	C11	N1	2.2(4)	C34	C29	C30	C31	0.3(5)
C12	C13	C14	C15	-1.8(7)	C26	C23	C22	C21	161.6(2)
C12	C17	C16	C15	1.1(7)	C13	C12	C17	C16	-0.4(6)
C29	N3	C18	S2	179.8(2)	C30	C29	C34	C33	-1.6(5)
C29	N3	C18	N4	-0.9(4)	C30	C31	C32	C33	-0.5(7)
C29	C34	C33	C32	1.9(6)	C14	C15	C16	C17	-2.1(7)
C29	C30	C31	C32	0.8(6)	C31	C32	C33	C34	-0.8(7)
C19	N4	C18	S2	2.9(4)	C17	C12	C13	C14	0.8(6)
C19	N4	C18	N3	-176.4(2)	C8	C7	C4	C3	65.9(3)
C19	C24	C23	C25	-36.7(2)	C8	C7	C4	C5	176.6(3)
C19	C24	C23	C22	69.6(2)	C9	C7	C4	C3	-171.8(3)
C19	C24	C23	C26	-166.5(2)	C9	C7	C4	C5	-61.1(4)
C19	C20	C21	C22	71.1(3)	C16	C15	C14	C13	2.5(7)
C24	C19	C20	C25	31.8(2)	C6	C1	C7	C4	-54.0(3)
C24	C19	C20	C21	-75.4(2)	C6	C1	C7	C8	-173.9(3)
C24	C23	C22	C21	-74.5(3)	C6	C1	C7	C9	63.0(4)
C23	C25	C20	C19	-52.2(2)	C10	C1	C7	C4	-179.1(3)
C23	C25	C20	C21	56.3(2)	C10	C1	C7	C8	61.1(4)
C23	C22	C21	C20	2.7(3)	C10	C1	C7	C9	-62.0(5)

Table S13. Torsion Angles for **6.**

A	B	C	D	Angle/°	A	B	C	D	Angle/°
C11	N1	C3	C2	74.3(4)	C10	C1	C6	C5	160.5(4)

Table S14. Hydrogen Atom Coordinates (Å×10^4) and Isotropic Displacement Parameters (Å^2×10^3) for **6.**

Atom	*x*	*y*	*z*	U(eq)
H2B	2331.03	7003.45	2162.67	82
H1	3162.42	4560.96	4404.49	113
H4A	5171.53	6904.94	1810.25	59
H3A	9082.36	6108.77	2599.52	70
H1A	4299.29	3056.84	3156.75	63
H2	600.45	4047.7	2299.91	70
H2A	5854.73	5286.52	4599.11	68
H19	3895.51	4876.05	1368.08	51
H24	1346.33	5639.04	1497.7	53
H22A	253.86	4781.81	486.04	65
H22B	-3.3	5298.34	-304.1	65
H20	5068.35	5697.75	267.33	57
H21A	2891.27	4095.58	339.39	67
H21B	2677.1	4665.05	-435.49	67
H27A	2936.76	8495.62	241.04	100
H27B	4804.85	7954.43	256.74	100
H27C	3909.06	7889.13	945.87	100
H28A	1629.77	6251.27	-886.5	113
H28B	3359.48	6943.55	-897.35	113
H28C	1598.71	7553.69	-814.58	113
H3	6143.75	4915.92	3477.24	61
H34	7683.31	8219.69	1649.82	85
H26A	-72.31	7874.98	660.15	100
H26B	-1312.2	6828.47	621.46	100
H26C	-830.68	7325	-94.23	100
H15	2441.23	-365.15	1818.11	100
H4	7405.4	2927.8	3387.93	75
H13	397.75	1909.89	3005.36	98
H30	7679.38	7174.61	3682.43	92
H14	873.43	51.05	2727.53	114
H31	7070.11	9031.08	3990.66	112
H17	3257.52	2871.86	1547.24	91
H32	6724.23	10391.37	3115.41	112

Table S14. Hydrogen Atom Coordinates (Å×10^4) and Isotropic Displacement Parameters (Å^2×10^3) for **6.**

**Atom**	***x***	***y***	***z***	**U(eq)**
H33	6989.69	9984.06	1965.51	107
H8A	5552.36	1613.27	4878.21	135
H8B	5481.66	1516.84	4036.21	135
H8C	4480.91	2490.29	4349.66	135
H9A	9849.9	2419.73	4829.12	135
H9B	8810.76	1410.39	4420.91	135
H9C	8656.14	1669.24	5229.77	135
H16	3703.55	1044.93	1265.45	103
H6A	9848.12	4134.59	5195.89	127
H6B	8732.83	5198.69	4899.04	127
H10A	6940.54	4380.58	5980.3	192
H10B	8001.1	3258.74	6006.57	192
H10C	5927.17	3253.25	5787.07	192
H5A	9034.01	4739.05	3754.39	119
H5B	10078.13	3644.3	4048.21	119

Table S15. Cartesian Coordinates for Complex 1

6	0.166640000	1.603847000	-3.406366000
8	0.698585000	1.225284000	-2.140411000
1	0.743383000	1.149940000	-4.220590000
1	0.232605000	2.691018000	-3.481268000
1	-0.886151000	1.318159000	-3.480356000
6	-3.123563000	1.938797000	-1.062365000
6	-3.218573000	0.555379000	-1.708515000
8	-1.965636000	-0.125065000	-1.617570000
6	-1.487973000	-0.277344000	-0.330666000
6	-1.350350000	1.057265000	0.393683000
6	-2.704715000	1.765812000	0.405425000
8	-2.130616000	2.636448000	-1.787400000
1	-4.098504000	2.437393000	-1.121429000
1	-3.975320000	-0.052365000	-1.187753000
8	-3.526674000	0.702603000	-3.044993000

8	-2.437621000	-1.019827000	0.463818000
1	-0.542642000	-0.816910000	-0.394051000
8	-0.900641000	0.809505000	1.706415000
1	-0.659275000	1.675323000	-0.179718000
1	-3.449454000	1.150316000	0.928057000
8	-2.619519000	3.058517000	0.961573000
6	-2.384679000	-2.345598000	0.540989000
6	-3.649770000	-2.815690000	1.297564000
7	-1.428150000	-3.056264000	0.135789000
17	-3.786539000	-4.587497000	1.284934000
17	-3.485144000	-2.252798000	2.983114000
17	-5.096908000	-2.105682000	0.559547000
6	0.350594000	1.229109000	2.077054000
6	1.356590000	1.602247000	1.186237000
6	0.590853000	1.218291000	3.452992000
6	1.842363000	1.576030000	3.935452000
6	2.600411000	1.985972000	1.689700000
6	2.855556000	1.966930000	3.056666000
6	-1.935466000	3.979143000	-1.562509000
6	-2.971124000	4.906205000	-1.636930000
6	-0.626227000	4.390087000	-1.320720000
6	-0.356423000	5.742967000	-1.138705000
6	-1.386963000	6.679272000	-1.189950000
6	-2.689643000	6.254581000	-1.441095000
6	-2.337875000	3.206032000	2.300576000
6	-1.437330000	4.216436000	2.626736000
6	-2.946116000	2.444870000	3.297293000
6	-2.636055000	2.702406000	4.629275000
6	-1.735622000	3.710244000	4.969293000
6	-1.141289000	4.466721000	3.962712000
6	-3.357664000	-0.483637000	-3.828376000
6	-4.019269000	-1.712908000	-3.250487000
6	-5.410865000	-1.838957000	-3.253008000

6	-6.016589000	-2.994527000	-2.768803000
6	-5.232838000	-4.036261000	-2.275019000
6	-3.847035000	-3.906651000	-2.246374000
6	-3.241149000	-2.745626000	-2.723701000
1	-1.592609000	-4.047597000	0.292807000
1	1.212284000	1.572777000	0.112580000
1	-0.222805000	0.940206000	4.114979000
1	2.022258000	1.565471000	5.006027000
1	3.364468000	2.280330000	0.978191000
1	3.829026000	2.260086000	3.436107000
1	-3.983499000	4.577038000	-1.845664000
1	0.156203000	3.636945000	-1.271840000
1	0.663128000	6.063667000	-0.949217000
1	-1.176416000	7.732789000	-1.040784000
1	-3.495938000	6.979316000	-1.492283000
1	-0.985780000	4.782189000	1.817597000
1	-3.641313000	1.652759000	3.040914000
1	-3.107652000	2.110029000	5.407358000
1	-1.500894000	3.903410000	6.010604000
1	-0.435323000	5.251313000	4.215180000
1	-2.289152000	-0.674329000	-3.969418000
1	-3.800210000	-0.229694000	-4.794435000
1	-6.016871000	-1.029512000	-3.652897000
1	-7.097863000	-3.088119000	-2.785209000
1	-5.702746000	-4.941138000	-1.902284000
1	-3.232525000	-4.713047000	-1.856909000
1	-2.159037000	-2.636769000	-2.687624000
1	0.711410000	0.244453000	-2.134648000
6	6.356666000	-1.125207000	-3.007542000
6	6.289987000	0.387152000	-3.365565000
6	5.685953000	-1.170915000	-1.619796000
6	4.185367000	-0.925810000	-1.884417000
6	4.128057000	0.620188000	-2.210211000

6	5.620175000	1.037210000	-2.130549000
6	6.176603000	0.150940000	-0.970638000
6	5.821617000	2.534989000	-2.021923000
6	7.699822000	0.218057000	-0.808016000
6	5.574701000	0.443702000	0.408805000
1	7.387318000	-1.483624000	-2.960000000
1	5.830272000	-1.753848000	-3.730193000
1	5.711789000	0.579367000	-4.274684000
1	7.286281000	0.808122000	-3.528950000
1	5.879488000	-2.086272000	-1.049798000
7	3.350714000	-1.329601000	-0.768838000
1	3.839796000	-1.504349000	-2.742787000
8	3.331577000	1.365043000	-1.326748000
1	3.759676000	0.758326000	-3.236422000
1	5.303743000	2.934825000	-1.146951000
1	6.886252000	2.781348000	-1.951459000
1	5.417860000	3.042227000	-2.904734000
1	8.253466000	0.056461000	-1.734075000
1	7.992821000	1.196423000	-0.413431000
1	8.029720000	-0.536668000	-0.085796000
1	5.863710000	-0.340666000	1.119926000
1	5.966523000	1.391993000	0.794873000
1	4.489364000	0.536438000	0.395936000
6	2.092627000	-1.768966000	-0.873320000
7	1.464802000	-2.118117000	0.268839000
16	1.263033000	-1.908635000	-2.367415000
6	1.932218000	-1.836705000	1.622185000
6	3.188451000	-2.607522000	1.996804000
6	4.110211000	-2.013679000	2.861650000
6	5.261974000	-2.698184000	3.242374000
6	5.502803000	-3.979872000	2.753996000
6	4.589235000	-4.573224000	1.884885000
6	3.436967000	-3.890218000	1.506867000

1	3.776776000	-1.319503000	0.149064000
1	2.427924000	1.417991000	-1.694597000
1	0.526593000	-2.496799000	0.143633000
6	0.785442000	-2.136322000	2.588319000
1	2.167507000	-0.764990000	1.692193000
1	3.921676000	-1.003814000	3.223680000
1	5.974341000	-2.227062000	3.912231000
1	6.403116000	-4.512228000	3.043130000
1	4.778490000	-5.568318000	1.495070000
1	2.731754000	-4.339143000	0.812505000
1	0.517380000	-3.197097000	2.539553000
1	-0.091661000	-1.528260000	2.341386000
1	1.095256000	-1.901086000	3.608394000

Table S16. Cartesian Coordinates for Complex 2

6	-0.490659000	1.399069000	-3.450178000
8	0.401135000	1.215485000	-2.366362000
1	-0.071933000	2.077636000	-4.204859000
1	-1.415475000	1.821962000	-3.052389000
1	-0.734797000	0.445153000	-3.933005000
6	-3.570931000	1.299209000	-0.481876000
6	-3.400730000	-0.025656000	-1.218357000
8	-2.030150000	-0.260365000	-1.525310000
6	-1.183244000	-0.270828000	-0.425360000
6	-1.258087000	1.032597000	0.370260000
6	-2.705220000	1.298071000	0.777748000
8	-3.130717000	2.363235000	-1.306321000
1	-4.625015000	1.432355000	-0.212883000
1	-3.762490000	-0.856977000	-0.591915000
8	-4.109996000	0.050526000	-2.403397000
8	-1.586490000	-1.292184000	0.503205000

1	-0.182469000	-0.476209000	-0.800040000
8	-0.486792000	0.921848000	1.547319000
1	-0.917694000	1.846797000	-0.276059000
1	-3.050288000	0.506996000	1.455093000
8	-2.829559000	2.571264000	1.373562000
6	-1.190884000	-2.547456000	0.270880000
6	-1.870692000	-3.449520000	1.324857000
7	-0.392260000	-2.879710000	-0.641681000
17	-1.448579000	-5.158038000	1.073908000
17	-1.321270000	-2.944873000	2.942506000
17	-3.632874000	-3.250628000	1.195491000
6	0.722905000	1.554227000	1.650534000
6	1.487446000	1.976471000	0.563700000
6	1.186474000	1.721289000	2.958529000
6	2.417840000	2.320262000	3.181943000
6	2.719807000	2.587067000	0.809792000
6	3.191212000	2.761604000	2.106916000
6	-4.032044000	3.370743000	-1.558260000
6	-5.246336000	3.108339000	-2.188034000
6	-3.672967000	4.667214000	-1.201329000
6	-4.542000000	5.715831000	-1.484839000
6	-5.764429000	5.469016000	-2.106954000
6	-6.112542000	4.165760000	-2.453311000
6	-2.939442000	2.689464000	2.732517000
6	-3.470476000	3.902328000	3.177308000
6	-2.554368000	1.711740000	3.650651000
6	-2.716703000	1.961595000	5.013012000
6	-3.243345000	3.165734000	5.465665000
6	-3.615503000	4.137405000	4.537186000
6	-3.858845000	-1.010078000	-3.325808000
6	-4.038967000	-2.388133000	-2.736150000
6	-5.299652000	-2.823920000	-2.319041000
6	-5.469302000	-4.108394000	-1.812639000

6	-4.376890000	-4.970207000	-1.718176000
6	-3.115002000	-4.534375000	-2.110223000
6	-2.945442000	-3.243581000	-2.608843000
1	-0.216117000	-3.882113000	-0.644646000
1	1.145131000	1.835438000	-0.458003000
1	0.555565000	1.387063000	3.775626000
1	2.770126000	2.452460000	4.200180000
1	3.318546000	2.922130000	-0.032325000
1	4.149798000	3.239467000	2.276782000
1	-5.480559000	2.087219000	-2.472302000
1	-2.724531000	4.821435000	-0.698500000
1	-4.266878000	6.729156000	-1.209745000
1	-6.441871000	6.288863000	-2.321111000
1	-7.060520000	3.969421000	-2.944274000
1	-3.765042000	4.635856000	2.434221000
1	-2.102554000	0.785645000	3.316554000
1	-2.416469000	1.198869000	5.725003000
1	-3.361109000	3.348072000	6.528253000
1	-4.029519000	5.082723000	4.873490000
1	-2.847908000	-0.907764000	-3.730727000
1	-4.578015000	-0.840691000	-4.131136000
1	-6.149375000	-2.150951000	-2.404145000
1	-6.453338000	-4.442182000	-1.498948000
1	-4.508574000	-5.974122000	-1.326950000
1	-2.260527000	-5.199711000	-2.028765000
1	-1.958069000	-2.890222000	-2.894050000
1	1.087769000	0.586992000	-2.646608000
6	1.832964000	-1.572339000	2.046930000
6	2.109383000	-3.093699000	1.888287000
6	3.074553000	-0.926552000	1.395551000
6	2.884425000	-1.128163000	-0.118911000
6	3.158223000	-2.677479000	-0.295423000
6	3.466564000	-3.147452000	1.150184000

6	4.206428000	-1.920742000	1.769158000
6	4.166027000	-4.490724000	1.212174000
6	4.434494000	-2.013345000	3.282486000
6	5.572908000	-1.621489000	1.135563000
1	1.744684000	-1.278358000	3.095759000
1	0.908564000	-1.248522000	1.558163000
1	1.328191000	-3.607754000	1.319033000
1	2.176205000	-3.598437000	2.856749000
1	3.238167000	0.114444000	1.688729000
7	3.777188000	-0.281228000	-0.899831000
1	1.859439000	-0.898181000	-0.425652000
8	4.221465000	-2.989781000	-1.162554000
1	2.236585000	-3.149324000	-0.658046000
1	5.106318000	-4.467925000	0.656857000
1	4.369273000	-4.772898000	2.250503000
1	3.539190000	-5.272004000	0.769253000
1	3.528939000	-2.217335000	3.855136000
1	5.158661000	-2.802650000	3.509521000
1	4.852138000	-1.069309000	3.649741000
1	5.898774000	-0.609110000	1.412228000
1	6.322647000	-2.316963000	1.527621000
1	5.571586000	-1.724897000	0.050946000
6	3.815327000	-0.258377000	-2.234234000
7	4.830046000	0.420040000	-2.815877000
16	2.661542000	-1.044603000	-3.223096000
6	6.028241000	0.893286000	-2.135399000
6	5.807905000	2.213971000	-1.415685000
6	6.349283000	2.421917000	-0.147802000
6	6.219651000	3.659967000	0.480279000
6	5.541498000	4.695545000	-0.154996000
6	4.992807000	4.490733000	-1.421270000
6	5.123118000	3.255779000	-2.046630000
1	4.407828000	0.317806000	-0.379240000

1	3.892037000	-2.850015000	-2.063165000
1	4.826533000	0.376028000	-3.825003000
6	7.150570000	1.022917000	-3.165146000
1	6.319717000	0.134703000	-1.394521000
1	6.883450000	1.613289000	0.347041000
1	6.645397000	3.811696000	1.467286000
1	5.432778000	5.657010000	0.335760000
1	4.453905000	5.292339000	-1.915572000
1	4.678722000	3.086976000	-3.023820000
1	6.879138000	1.761477000	-3.926186000
1	7.343925000	0.062346000	-3.649311000
1	8.064927000	1.364854000	-2.677325000

Table S17. Cartesian Coordinates for **Complex 3**

6	-0.002498000	-3.487643000	0.010321000
8	-0.291197000	-2.136223000	-0.341814000
1	0.488679000	-4.001745000	-0.817524000
1	-0.971173000	-3.941551000	0.220500000
1	0.645735000	-3.552733000	0.891640000
6	3.645394000	-1.365083000	0.170284000
6	3.033492000	-1.296851000	-1.223052000
8	1.785738000	-1.950904000	-1.190217000
6	0.808127000	-1.288502000	-0.429678000
6	1.307763000	-0.887110000	0.987380000
6	2.789140000	-0.493447000	1.071737000
8	3.593632000	-2.702401000	0.626256000
1	4.675398000	-0.990885000	0.158097000
1	2.877301000	-0.241173000	-1.520373000
8	3.857894000	-1.950830000	-2.114332000
1	0.456740000	-0.381403000	-0.945330000
8	0.552410000	0.261941000	1.406585000
1	1.139904000	-1.705707000	1.694832000

1	2.896927000	0.543309000	0.727359000
8	3.275685000	-0.634757000	2.394925000
6	-0.444023000	0.147417000	2.355643000
6	-1.410001000	-0.855053000	2.313336000
6	-0.472818000	1.143497000	3.329331000
6	-1.497536000	1.149842000	4.268495000
6	-2.411057000	-0.851643000	3.285334000
6	-2.467821000	0.148351000	4.253383000
6	4.800183000	-3.340797000	0.803357000
6	5.682565000	-2.924178000	1.797092000
6	5.090482000	-4.435855000	-0.003393000
6	6.279811000	-5.129931000	0.195043000
6	7.175272000	-4.722912000	1.182035000
6	6.875166000	-3.619273000	1.977495000
6	2.953499000	0.338946000	3.314187000
6	2.396837000	-0.080609000	4.519560000
6	3.239718000	1.686190000	3.095209000
6	2.934335000	2.616014000	4.085307000
6	2.374495000	2.206776000	5.294975000
6	2.115037000	0.855405000	5.510121000
6	3.298839000	-2.077402000	-3.427663000
6	2.821535000	-0.760139000	-3.989360000
6	3.744335000	0.175542000	-4.465992000
6	3.313841000	1.401652000	-4.959883000
6	1.951446000	1.703611000	-4.978527000
6	1.026503000	0.788876000	-4.481443000
6	1.463938000	-0.437266000	-3.980397000
1	-1.408479000	-1.588143000	1.512519000
1	0.316882000	1.890729000	3.345237000
1	-1.520988000	1.927073000	5.025773000
1	-3.161693000	-1.634180000	3.268562000
1	-3.258303000	0.142168000	4.996614000
1	5.411244000	-2.074563000	2.416466000

1	4.380990000	-4.709239000	-0.776417000
1	6.512319000	-5.986263000	-0.429986000
1	8.103927000	-5.263779000	1.330624000
1	7.567948000	-3.302233000	2.750580000
1	2.191784000	-1.136817000	4.657557000
1	3.691311000	2.007915000	2.162725000
1	3.149052000	3.665258000	3.909253000
1	2.148009000	2.936412000	6.065169000
1	1.678290000	0.526061000	6.447391000
1	2.475169000	-2.796761000	-3.403371000
1	4.112050000	-2.486707000	-4.031233000
1	4.804090000	-0.066724000	-4.450464000
1	4.035740000	2.121026000	-5.333752000
1	1.613577000	2.654899000	-5.379200000
1	-0.034998000	1.018302000	-4.466029000
1	0.743516000	-1.141321000	-3.570092000
8	1.692241000	2.615477000	0.700700000
6	1.624678000	2.753353000	-0.619644000
6	2.804838000	3.620381000	-1.111684000
7	0.756977000	2.179226000	-1.331812000
17	2.498496000	4.258032000	-2.734142000
17	3.099142000	4.973790000	-0.001411000
17	4.238055000	2.548075000	-1.146176000
1	0.871243000	2.326203000	-2.334282000
1	1.143919000	1.839091000	0.972576000
6	-6.850852000	-1.928854000	-2.042221000
6	-6.337697000	-3.384872000	-1.846665000
6	-6.075057000	-1.147192000	-0.961388000
6	-4.624671000	-1.072000000	-1.475112000
6	-4.101149000	-2.546209000	-1.276232000
6	-5.332875000	-3.271821000	-0.675861000
6	-6.008576000	-2.170807000	0.204023000
6	-4.992993000	-4.587578000	-0.004702000

6	-7.389706000	-2.556450000	0.747069000
6	-5.170245000	-1.722936000	1.406697000
1	-7.929455000	-1.855940000	-1.886758000
1	-6.643470000	-1.540959000	-3.042820000
1	-5.856743000	-3.786305000	-2.743661000
1	-7.151719000	-4.068536000	-1.588587000
1	-6.499288000	-0.165891000	-0.717199000
7	-3.848277000	-0.074214000	-0.765796000
1	-4.589963000	-0.804504000	-2.534267000
8	-2.984118000	-2.644185000	-0.429534000
1	-3.854339000	-2.976770000	-2.256381000
1	-4.264741000	-4.438059000	0.796134000
1	-5.890493000	-5.056819000	0.411942000
1	-4.553538000	-5.284594000	-0.726041000
1	-8.092091000	-2.890754000	-0.017690000
1	-7.293947000	-3.360343000	1.484349000
1	-7.838454000	-1.697558000	1.258200000
1	-5.567772000	-0.786713000	1.822498000
1	-5.231237000	-2.478531000	2.199020000
1	-4.117411000	-1.592069000	1.161782000
6	-2.682674000	0.421634000	-1.208159000
7	-2.138042000	1.446361000	-0.515616000
16	-1.923868000	-0.181254000	-2.606585000
6	-2.677934000	2.064130000	0.690984000
6	-4.120709000	2.520146000	0.535359000
6	-5.007405000	2.349056000	1.599064000
6	-6.333581000	2.764219000	1.487422000
6	-6.779832000	3.351355000	0.307353000
6	-5.896636000	3.523107000	-0.758560000
6	-4.573803000	3.109707000	-0.646046000
1	-4.251402000	0.300704000	0.081433000
1	-2.233594000	-2.197596000	-0.860839000
1	-1.191623000	1.695135000	-0.800923000

6	-1.791875000	3.258849000	1.041298000
1	-2.645715000	1.345336000	1.523204000
1	-4.652025000	1.876993000	2.513706000
1	-7.016706000	2.622742000	2.318903000
1	-7.813155000	3.669246000	0.214031000
1	-6.244095000	3.972758000	-1.683219000
1	-3.887858000	3.214000000	-1.482760000
1	-1.850914000	4.012753000	0.250457000
1	-0.748877000	2.954042000	1.157525000
1	-2.126732000	3.705125000	1.979915000

Table S18. Cartesian Coordinates for **Complex 4**

6	-1.784353000	0.258422000	4.848757000
8	-1.242843000	1.086178000	3.835328000
1	-0.997466000	-0.296032000	5.374423000
1	-2.505895000	-0.452077000	4.429786000
1	-2.293820000	0.906574000	5.566157000
1	-0.896672000	0.493738000	3.145488000
6	5.228032000	-1.063876000	0.359874000
6	5.295847000	-0.607349000	-1.125899000
6	4.810530000	0.229826000	1.078603000
6	3.344209000	0.467953000	0.644148000
6	3.478166000	1.009768000	-0.823930000
6	5.000610000	0.916791000	-1.074248000
6	5.614647000	1.310804000	0.307758000
6	5.458688000	1.691865000	-2.292801000
6	7.133588000	1.101808000	0.384439000
6	5.363954000	2.757850000	0.755587000
1	6.193092000	-1.427776000	0.719506000
1	4.499901000	-1.862459000	0.518667000
1	4.564490000	-1.130745000	-1.750589000
1	6.280987000	-0.791252000	-1.565282000
1	4.929742000	0.214635000	2.165763000

7	2.615183000	1.414911000	1.458592000
1	2.797831000	-0.479016000	0.666855000
8	2.982989000	2.328612000	-0.964642000
1	2.938669000	0.355944000	-1.521479000
1	5.183765000	2.746229000	-2.208050000
1	6.543644000	1.615343000	-2.418628000
1	4.989011000	1.294354000	-3.199523000
1	7.465003000	0.123090000	0.035598000
1	7.648321000	1.858673000	-0.216509000
1	7.473529000	1.220669000	1.419061000
1	5.619239000	2.866176000	1.815749000
1	6.012564000	3.436392000	0.190602000
1	4.343302000	3.100097000	0.602250000
6	1.903245000	1.100475000	2.554361000
7	1.370134000	2.139506000	3.236122000
16	1.661593000	-0.496716000	3.087099000
6	1.510852000	3.541809000	2.868526000
6	0.778665000	3.888221000	1.578060000
6	-0.506707000	3.386144000	1.368852000
6	-1.198403000	3.686532000	0.199880000
6	-0.618251000	4.513956000	-0.761850000
6	0.662779000	5.019795000	-0.557855000
6	1.361311000	4.701999000	0.606702000
1	2.529758000	2.339688000	1.050952000
1	0.636171000	1.882623000	3.892243000
1	2.581657000	3.748261000	2.729419000
6	0.989614000	4.387278000	4.029832000
1	-0.941539000	2.724943000	2.114834000
1	-2.179919000	3.251333000	0.028733000
1	-1.158878000	4.744863000	-1.676296000
1	1.128109000	5.649730000	-1.309285000
1	2.372185000	5.077049000	0.752306000
1	1.547075000	4.168097000	4.943551000

1	-0.069398000	4.167695000	4.199532000
1	1.085815000	5.449147000	3.795546000
6	-2.135293000	-1.700965000	0.982899000
6	-2.538352000	-0.233399000	0.943213000
8	-1.364478000	0.586267000	1.005845000
6	-0.501470000	0.416178000	-0.064605000
6	-0.037587000	-1.033911000	-0.202972000
6	-1.240620000	-1.979419000	-0.227468000
8	-1.425535000	-1.896904000	2.182704000
1	-3.045399000	-2.309617000	0.941868000
1	-3.060063000	-0.005280000	0.001949000
8	-3.328058000	0.034081000	2.038585000
8	-1.243087000	0.752800000	-1.252592000
8	0.670033000	-1.085861000	-1.425775000
1	0.589761000	-1.250351000	0.669913000
1	-1.818146000	-1.797915000	-1.143429000
8	-0.802261000	-3.319566000	-0.160035000
6	-0.709642000	1.464432000	-2.236109000
6	-1.811556000	1.646205000	-3.307719000
7	0.473878000	1.889395000	-2.285055000
17	-1.279688000	2.747642000	-4.595824000
17	-2.149078000	0.039219000	-4.004842000
17	-3.280091000	2.294831000	-2.544249000
6	1.655625000	-2.017989000	-1.633745000
6	2.158629000	-2.882978000	-0.664722000
6	2.146181000	-2.053281000	-2.941880000
6	3.137022000	-2.962126000	-3.284915000
6	3.157465000	-3.787200000	-1.027881000
6	3.651327000	-3.835147000	-2.326392000
6	-1.451644000	-3.106747000	2.828036000
6	-2.142832000	-4.235673000	2.389865000
6	-0.720166000	-3.146902000	4.016905000
6	-0.694006000	-4.311490000	4.771307000

6	-1.394053000	-5.443073000	4.354391000
6	-2.109854000	-5.395183000	3.164194000
6	-0.939240000	-4.145244000	-1.246121000
6	-1.033072000	-5.506819000	-0.956255000
6	-0.942004000	-3.698308000	-2.566693000
6	-1.062889000	-4.630652000	-3.595310000
6	-1.169096000	-5.989376000	-3.319250000
6	-1.146685000	-6.422429000	-1.994201000
6	-3.803185000	1.372850000	2.134644000
6	-4.717147000	1.772034000	0.996818000
6	-5.520867000	0.828838000	0.352441000
6	-6.359663000	1.210093000	-0.689984000
6	-6.418191000	2.543974000	-1.089968000
6	-5.635684000	3.494429000	-0.438262000
6	-4.786196000	3.106877000	0.596089000
1	0.646215000	2.433419000	-3.127434000
1	1.772063000	-2.884084000	0.348435000
1	1.725334000	-1.362521000	-3.666011000
1	3.509900000	-2.987142000	-4.303792000
1	3.546690000	-4.463254000	-0.273297000
1	4.424711000	-4.547336000	-2.591675000
1	-2.671918000	-4.243052000	1.445053000
1	-0.172005000	-2.257712000	4.311978000
1	-0.118953000	-4.333836000	5.691440000
1	-1.373254000	-6.351843000	4.945815000
1	-2.649998000	-6.271148000	2.817892000
1	-1.011105000	-5.814682000	0.084328000
1	-0.817285000	-2.644575000	-2.792732000
1	-1.058874000	-4.283470000	-4.623717000
1	-1.259364000	-6.706231000	-4.128028000
1	-1.220864000	-7.480854000	-1.765819000
1	-2.957784000	2.062899000	2.216150000
1	-4.340348000	1.398906000	3.087376000

1	-5.475507000	-0.208956000	0.671646000
1	-6.970436000	0.466122000	-1.191206000
1	-7.071543000	2.840828000	-1.903902000
1	-5.678706000	4.536161000	-0.740086000
1	-4.165818000	3.847760000	1.094763000
1	0.337418000	1.107226000	0.057400000
1	2.067995000	2.255694000	-1.290127000

1. Periasamy, M.; Sanjeevakumar, N.; Obula Reddy, P., Convenient Methods to Access Chiral Camphanyl Amine Derivatives by -Sodium Borohydride Reduction of d-(–)-Camphorquinone Imines. *Synthesis* **2012,** *44* (20), 3185-3190.
2. Santacruz, E.; Huelgas, G.; Angulo, S. K.; Mastranzo, V. M.; Hernández-Ortega, S.; Aviña, J. A.; Juaristi, E.; Parrodi, C. A. d.; Walsh, P. J., Catalytic asymmetric hydrosilylation of acetophenone with new chiral thiourea ligands containing the (S)-α-phenylethyl group. *Tetrahedron: Asymmetry* **2009,** *20* (24), 2788-2794.
